# Structural Insights for Core Scaffold and Substrate Specificity of B1, B2, and B3 Metallo-β-Lactamases

**DOI:** 10.3389/fmicb.2021.752535

**Published:** 2022-01-13

**Authors:** Yeongjin Yun, Sangjun Han, Yoon Sik Park, Hyunjae Park, Dogyeong Kim, Yeseul Kim, Yongdae Kwon, Sumin Kim, Jung Hun Lee, Jeong Ho Jeon, Sang Hee Lee, Lin-Woo Kang

**Affiliations:** ^1^Department of Biological Sciences, Konkuk University, Seoul, South Korea; ^2^National Leading Research Laboratory of Drug Resistance Proteomics, Department of Biological Sciences, Myongji University, Yongin, South Korea

**Keywords:** metallo-β-lactamase (MBL), β-lactams, metal coordination, substrate specificity, β-lactamase inhibitor, antibiotic resistance

## Abstract

Metallo-β-lactamases (MBLs) hydrolyze almost all β-lactam antibiotics, including penicillins, cephalosporins, and carbapenems; however, no effective inhibitors are currently clinically available. MBLs are classified into three subclasses: B1, B2, and B3. Although the amino acid sequences of MBLs are varied, their overall scaffold is well conserved. In this study, we systematically studied the primary sequences and crystal structures of all subclasses of MBLs, especially the core scaffold, the zinc-coordinating residues in the active site, and the substrate-binding pocket. We presented the conserved structural features of MBLs in the same subclass and the characteristics of MBLs of each subclass. The catalytic zinc ions are bound with four loops from the two central β-sheets in the conserved αβ/βα sandwich fold of MBLs. The three external loops cover the zinc site(s) from the outside and simultaneously form a substrate-binding pocket. In the overall structure, B1 and B2 MBLs are more closely related to each other than they are to B3 MBLs. However, B1 and B3 MBLs have two zinc ions in the active site, while B2 MBLs have one. The substrate-binding pocket is different among all three subclasses, which is especially important for substrate specificity and drug resistance. Thus far, various classes of β-lactam antibiotics have been developed to have modified ring structures and substituted R groups. Currently available structures of β-lactam-bound MBLs show that the binding of β-lactams is well conserved according to the overall chemical structure in the substrate-binding pocket. Besides β-lactam substrates, B1 and cross-class MBL inhibitors also have distinguished differences in the chemical structure, which fit well to the substrate-binding pocket of MBLs within their inhibitory spectrum. The systematic structural comparison among B1, B2, and B3 MBLs provides in-depth insight into their substrate specificity, which will be useful for developing a clinical inhibitor targeting MBLs.

## Introduction

The increasing incidence of multidrug-resistant (MDR) bacteria is a global health concern (Laxminarayan et al., 2013; Berendonk et al., 2015; Lee et al., 2016). β-lactams constitute 60% of current antibiotics; thus far, they have been the most applicable and useful class of antibiotics (Ozturk et al., 2015). However, the frequent clinical use of β-lactams has caused selective pressure, resulting in the rapid appearance of bacterial resistance to β-lactams. The most common mechanism of β-lactam resistance among MDR bacteria is the production of β-lactamases, which hydrolyze β-lactams into inactive forms (Paterson et al., 2020; Bahr et al., 2021). The evolution and catalytic mechanisms of various β-lactamases have been studied (Hall and Barlow, 2004; Sidjabat et al., 2018; Lee et al., 2019; Park et al., 2020; Pedroso et al., 2020). β-lactamases can be divided into serine β-lactamases and metallo- β-lactamases (MBLs). MBLs hydrolyze most β-lactams, including last resort antibiotics carbapenems. There are currently no effective and clinically available inhibitors against MBLs (Fisher et al., 2005). MBLs are further classified into the B1, B2, and B3 subclasses depending on their sequence, structure, and zinc ion site(s) and have diverse substrate profile or specificity for β-lactams (Crowder et al., 2006; Palacios et al., 2019; Behzadi et al., 2020; Park et al., 2020).

The substrate profile of MBLs is related to the antimicrobial susceptibility of MBL producers and is essential for the adequate treatment of patients with MBL-producing MDR bacteria (Lutgring et al., 2020). However, the main interest of antibiotic resistance study has been the efficacy and effectiveness of specific antibiotics and inhibitors on MDR bacteria in clinical use. The previous study of the substrate profile showed that B1 and B3 MBLs have a broad substrate spectrum, and B2 MBLs degrade only carbapenems (Bahr et al., 2021). Even in a subclass, there are many different types of MBLs and a growing number of variants, which also could have diverse hydrolytic activities on β-lactams; thus far, 710 MBLs of 509 B1, 22 B2, and 179 B3 members were reported (Naas et al., 2017). Independent research groups have studied the substrate profile and enzyme kinetics of MBLs with varied assay conditions. There are only limited numbers of MBL structures available for the study of structure-function relationships. The complexity and insufficiency of MBL data have prohibited the systematic study of the structure-based substrate specificity of MBLs. Herein, we compared several tens of B1, B2, and B3 MBLs in sequence and structure and proposed structural insights on the substrate specificity of MBLs. The specificity of the substrate-binding pocket of MBLs was also verified by B1 and cross-class MBL inhibitors binding to the same substrate-binding pocket. The cross-class inhibitors showed the complementary chemical structures to fit into the varied substrate-binding pockets of different subclasses of MBLs. The structural insights of MBLs will provide a valuable platform to understand the structure-function relationships of current and newly found putative MBLs and develop a broad-spectrum MBL inhibitor.

### Scaffold of Metallo-β-Lactamases

**FIGURE 1:**
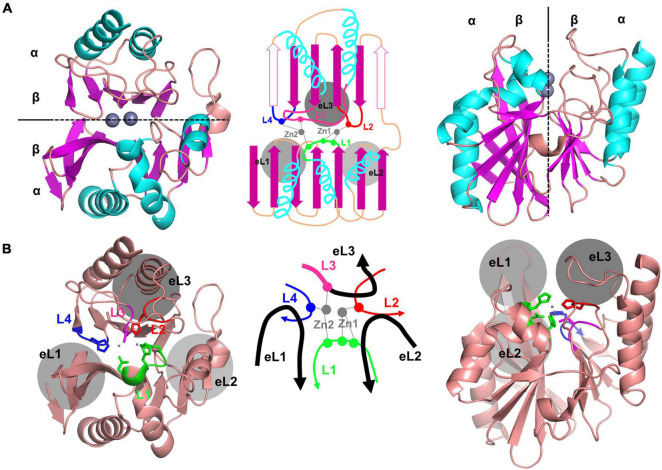
The crystal structure of NDM-1, showing the representative core scaffold of MBLs. **(A)** The αβ/βα sandwich fold of NDM-1, which is the core scaffold of MBLs, is shown at the top (left) and side (right). The schematic representation is shown in the middle. The central two β-sheets and the five α-helices in the main scaffold are shown in purple and cyan, respectively. The two zinc ions are shown in gray. Certain β-strands in the second β-sheet exist as α-helices in some MBLs, which are shown as open purple arrows in the schematic representation (middle). **(B)** The overall structure of NMD-1 with the zinc-coordinating central loops L1–4 and the substrate-binding pocket forming the external loops eL1–3. The schematic representation of the central and external loops with zinc ions is shown in the middle. L3 is the N-terminal part of eL3.

The amino acid sequences of MBLs varied; the sequence identity among them could be as low as 10%. Within the same subclass, the B1, B2, and B3 MBLs had average sequence identities of 31.8, 60.2, and 33.0%, respectively (Supplementary Table 1). Although there was low sequence conservation, the MBL scaffold had the distinctive αβ/βα sandwich fold (Figure 1A) and was well conserved, as indicated by an RMSD value of 1.77 Å in approximately 220 residues (Supplementary Table 2). In the superimposed crystal structures of MBLs, there were conserved secondary structures of 5 α-helices and 13 β-strands as the core scaffold. The four loops of L1–4 coordinated the catalytic zinc ion(s), and the three external loops (eLs) eL1–3 formed the substrate-binding pocket (Figure 1B). The overall structure of the B1, B2, and B3 MBLs showed RMSD values of 1.45 in 205 residues, 0.65 in 223 residues, and 1.51 in 231 residues, respectively, among the members of the same subclass (Supplementary Tables 3–5).

**FIGURE 2:**
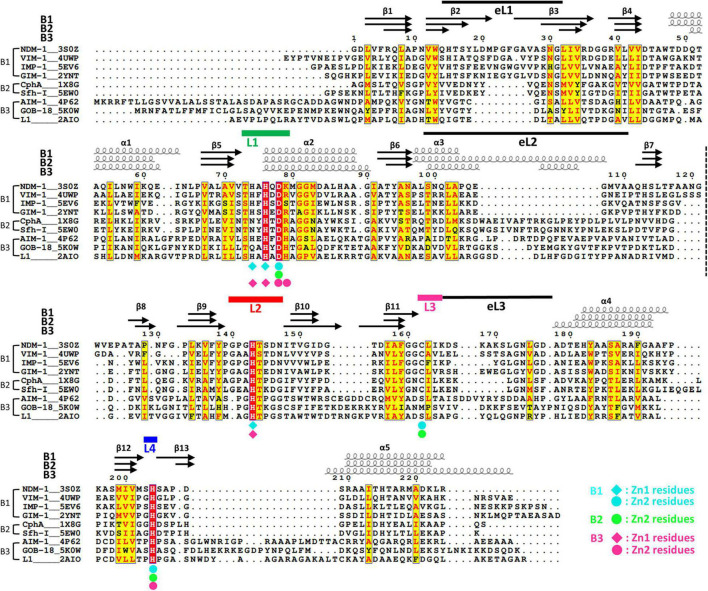
The structural sequence alignment of MBLs. The secondary structures of NDM-1, CphA, and AIM-1 are shown at the top. The labels B1, B2, and B3 show the representative member of each subclass. The numbering of secondary structures is based on the core scaffold of 13 β-strands and 5 α-helices. In some B3 MBLs, including AIM-1, an additional α-helix (α3′) exists after α3. The first and second halves of the αβ/βα sandwich fold are divided by a dashed line between the β7 and β8 strands. The zinc-coordinating residues of the B1, B2, and B3 subclasses are shown as diamonds and circles in cyan, green, and pink, respectively. The four zinc-coordinating loops L1, L2, L3, and L4 are shown as thick green, red, pink, and blue lines, respectively. The three external loops eL1, eL2, and eL3 are shown as thin black lines.

**FIGURE 3:**
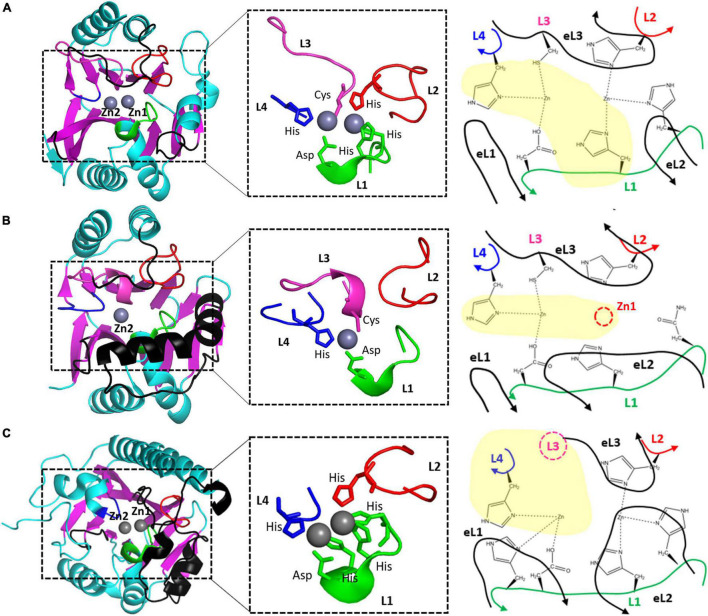
The zinc coordination of **(A)** NDM-1, **(B)** CphA, and **(C)** AIM-1 as the representative B1, B2, and B3 MBLs. The schematic representations of zinc coordination are shown on the right. The four zinc-coordinating loops L1, L2, L3, and L4 are shown as green, red, pink, and blue lines, respectively. The three external loops eL1, eL2, and eL3 are shown as black lines. L3 is the N-terminal part of eL3 and is shown in black. The missing Zn1 ion and Zn2-coordinating residue from L3 are shown as red and pink dashed circles, respectively.

The MBL structure can be divided into two parts at the interface between the two β-sheets (Figure 1A). The active site is located at the center between the two β-sheets, wherein the zinc ions are coordinated with various residues depending on each subclass (Figures 1B, 2; Ullah et al., 1998; Fonseca et al., 2011b; King and Strynadka, 2011; Pedroso et al., 2020). The zinc-coordinating residues come from the L1–4 loops protruding from the two β-sheets. The zinc ions directly coordinate a catalytic water molecule, which is deprotonated to a hydroxide ion to attack the β-lactam ring of the substrate (Supplementary Figure 1). The central L1–4 loops are surrounded by the external loops eL1–3, which form the substrate-binding pocket and play an important role in the substrate specificity of MBLs (Figures 1B, 3).

### Representative Metallo-β-Lactamases in Each Subclass

Although many MBLs from the three different subclasses have been studied in parallel, these comparisons were mainly related to the catalytic zinc ion(s) and the sequence and structure of the coordinating residues. There is only limited comparative information about the structure of the core scaffold, the substrate-binding pocket, and the relationship between drug resistance and structure. In this study, we performed a systematic comparison of the sequence and structure of MBLs, both with protein alone and in complex with substrate antibiotics. First, the sequences and structures of MBLs were compared within the same subclass. Second, the representative MBL structures from each subclass were compared with those from the other subclasses. Finally, the β-lactam or inhibitor-bound B1, B2, and B3 MBL structures were studied based on the characteristics of each subclass.

For structural comparison, 11, 2, and 8 MBLs were selected from the B1, B2, and B3 subclasses, respectively (Figure 2 and Supplementary Figures 2–4). These MBLs included the New Delhi metallo- β-lactamase (NDM-1) (PDB ID: 3S0Z, Guo et al., 2011), BlaB-1 (1M2X, Garcia-Saez et al., 2003a), VIM-2 (4NQ2, Aitha et al., 2014), DIM-1 (4ZEJ, Booth et al., 2015), IMP-1 (5EV6, Hinchliffe et al., 2016), TMB-1 (5MMD, Skagseth et al., 2017), SPS-1 (6CQS, Cheng et al., 2018), ECV-1 (6T5K, Frohlich et al., 2020), MYO-1 (6T5L, Frohlich et al., 2020), FIM-1 (6V3Q), and GIM-1 (2YNT, Borra et al., 2013) in B1; CphA (1X8G, Garau et al., 2005) and SfhI (5EW0, Hinchliffe et al., 2016) in B2; and Adelaide imipenemase (AIM-1) (4AWZ, Leiros et al., 2012), GOB-18 (5K0W, Moran-Barrio et al., 2016), FEZ-1 (1K07, Garcia-Saez et al., 2003b), Rm3 (5IQK, Salimraj et al., 2016), SMB-1 (3VPE, Wachino et al., 2013), L1 (2AIO, Spencer et al., 2005), BJP-1 (5NJW, Di Pisa et al., 2018), and LRA-12 (5AEB, Rodriguez et al., 2017) in B3. Among them, NDM-1 in the B1 subclass (Khan et al., 2017), CphA in the B2 subclass (Hernandez Valladares et al., 1997), and AIM-1 in the B3 subclass (Yong et al., 2012) were selected as the representative MBLs of each subclass for structural comparison (Figure 3). NDM-1 is found in the clinically important *Klebsiella pneumoniae*, *Enterobacter cloacae*, *Pseudomonas* spp., and *Acinetobacter baumannii*, and is mostly found in plasmids. NMD-1 hydrolyzes a wide range of β-lactams (Khan et al., 2017) and NDM-1 producers are resistant to imipenem, meropenem, ertapenem, gentamicin, amikacin, tobramycin, and ciprofloxacin; meanwhile, NDM-1 producers are susceptible to colistin and tigecycline (Kumarasamy et al., 2010). CphA was originally found in *Aeromonas hydrophila* and has a narrow substrate specificity for carbapenems (Hernandez Valladares et al., 1997). AIM-1 was found in *P. aeruginosa* and hydrolyzes a wide range of substrates, such as imipenem, meropenem, penicillin G, piperacillin, cephalothin, cefoxitin, and cefepime; however, it has no activity against aztreonam (Yong et al., 2012; Selleck et al., 2016).

When we performed structural sequence alignment, the internal sequence identity among MBLs within the same subclass was higher than that between the MBLs of different subclasses. NDM-1 was compared with 10 other MBLs in B1; CphA was compared with SfhI in B2: and AIM-1 was compared with seven other MBLs in B3 (Supplementary Table 1). The RMSD value was 1.45 Å in approximately 205 residues when comparing NDM-1 with the selected members in B1. The RMSD values comparing NDM-1 for B2 and B3 MBLs were 1.42 Å in approximately 195 residues and 2.25 Å in approximately 176 residues, respectively (Supplementary Tables 2–5). These results show that the overall scaffold is more similar between B1 and B2 MBLs than between B3 MBLs and the other two subclasses.

### B1 Subclass

Members of the B1 subclass exist in large numbers and contain many clinically important MDLs, such as NDMs, Verona integrin-encoded MBLs (VIMs), imipenemases (IMPs), and German imipenemases (GIMs). In the B1 subclass, 11 MBLs, including NDM-1 (PDB ID: 3S0Z, Guo et al., 2011), VIM-2 (4NQ2, Aitha et al., 2014), IMP-1 (5EV6, Hinchliffe et al., 2016), and GIM-1 (2YNT, Borra et al., 2013), were chosen as representative B1 MBLs (Supplementary Figure 2).

When the crystal structures of the representative B1 members were superimposed, the RMSD values among structures were between 1.06 and 1.76 Å in 205 residues. This finding shows that the overall scaffold is well conserved within the B1 subclass (Supplementary Table 3). The two central β-sheets in a core scaffold generally consist of seven β-strands and six β-strands in the first and second β-sheets, respectively (Figure 1A). Even though the terminal β-strands located at the ends of β-sheets are often changed to an α-helix or loop in certain members, the overall scaffold is well conserved (Figure 3A). The N-terminal sequences also varied; before the β1 strand, additional secondary structures could exist, such as an additional α-helix or β-strand (Supplementary Figure 5). Among the 13 β-strands, β2 and β3 are long, and β1 is only half the length of β2. The protruding tips of β2 and β3 of eL1 have a flexible conformation (Raczynska et al., 2020).

The two zinc ions are coordinated with four loops in the active site: short L1, long L2, extralong L3, and short L4 (Figure 3A). The first zinc ion, Zn1, is coordinated with three His residues (two from L1 and one from L2), and the second zinc ion, Zn2, is coordinated with Asp, Cys, and His residues from L1, L3, and L4, respectively. All six residues are strictly conserved in the sequences of B1 MBLs; among the 11 MBLs in B1, only SPS-1 loses one His residue in L1 (Supplementary Figure 2). All the zinc-coordinating residues exist at the tip of the secondary structures of the helix and strand; they are tightly wrapped in the center with three external loops (eL1–3) from the outside. The stable zinc-coordinating residues contain two metal ions at the catalytic positions.

**FIGURE 4:**
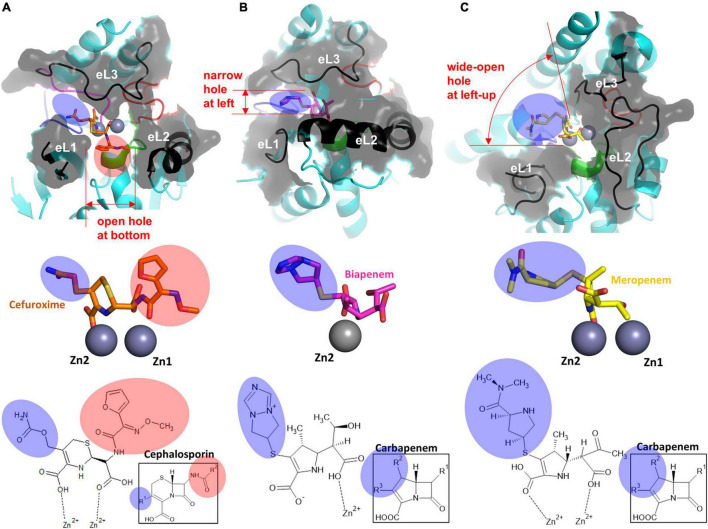
The structure of the substrate-binding pocket forming eL1–3 of each subclass. The surface representation of eL1, eL2, and eL3 of **(A)** cefuroxime-bound NDM-1 (PDB ID: 3SPU), **(B)** biapenem-bound CphA (PDB ID: 1X8I), and **(C)** meropenem-bound SMB-1 (PDB ID: 5AXO) is shown at the top. The enlarged view of the hydrolyzed β-lactams with the zinc ion(s) is shown in the middle. The zinc ion(s)-bound chemical structures of β-lactams, including the core structures of each β-lactam group, are shown at the bottom. The substituted R groups at the four-member β-lactam ring are shaded in pink, and those at the five or six-membered dihydropyrrole ring or dihydrothiazine ring next to the β-lactam ring are shaded in blue.

The three external loops eL1, eL2, and eL3, including L3, surround the zinc binding sites and form the substrate-binding pocket as three protruding fingers (Figure 1B). eL1 forms the left wall with long and flexible β2 and β3 (Figure 4A). At the bottom and right side of the pocket, eL2 provides a large hole in the central bottom of the pocket, which allows the flexible binding of bulky substrates. eL3 is extruded and shows a natural curvy-loop conformation to form the entire upper lip of the substrate-binding pocket.

### B2 Subclass

B2 MBLs, existing in 3% among all known MBLs, include CphA (Garau et al., 2005), SfhI from *Serratia fonticola* (Hinchliffe et al., 2016), ImiS from *Aeromonas sobria* (Walsh et al., 1996), and AsbM1 from *Aeromonas sobria* (Yang and Bush, 1996) and preferentially hydrolyze carbapenems (Fonseca et al., 2011a). Among them, only two crystal structures of CphA (PDB ID: 1X8G, Garau et al., 2005) and SfhI (5EW0, Hinchliffe et al., 2016) were determined, and the RMSD value between them was 0.65 (Supplementary Table 4). B2 MBLs have a zinc ion in the Zn2 site, which is coordinated with Asp, Cys, and His residues and loses the other zinc ions at the Zn1 site (Figure 3B and Supplementary Figure 3).

In the B2 subclass, both structures of CphA and SfhI showed a well-conserved core scaffold of MBLs with two central β-sheets of seven β-strands and six β-strands (Figure 3B). β2 and β3 are shorter in the B2 subclass compared with the B1 subclass in their lengths, and the resulting lengths of β1, β2, and β3 are similar (Figure 2 and Supplementary Figure 6). The helix α3 is long and bent in the middle, and the end of long α3 is positioned close to eL1 (Supplementary Figure 6).

CphA lost the Zn1 ion and maintained only the Zn2 ion. All three Zn2-coordinating residues in CphA of Asp, Cys, and His from L1, L3, and L4, respectively, are conserved in B2 MBLs (Supplementary Figure 3). In the Zn1 site, the first His residue among the three conserved His residues is changed to an Asn residue with a shorter side chain, which is insufficient to coordinate Zn1 compared to the canonical His residue (Figure 3B). The remaining two His residues were not sufficient to bind the Zn1 ion in CphA.

Although the overall structures of the zinc-coordinating L1–4 loops in the active site of CphA are conserved with NMD-1, all three external loops forming the substrate-binding pocket are different (Figure 4B). eL1 is shorter because of the shorter β2 and β3 and provides a shallow left boundary of the substrate-binding pocket. eL2, consisting of long and bent helix α3, forms a solid wall in the lower lip of the substrate-binding pocket, which could restrain substrate binding and accordingly affect the substrate specificity of CphA. eL3 of the upper lip of the substrate-binding pocket is slightly shorter than that found in B1 MBLs but adopts a similar conformation.

### B3 Subclass

B3 MBLs include SMB-1 (Wachino et al., 2013), AIM-1 (Leiros et al., 2012), L1 (Spencer et al., 2005), GOB-1 (Moran-Barrio et al., 2016), MIM-1 (Selleck et al., 2020), SAM-1 (Selleck et al., 2020), CSR-1 (Pedroso et al., 2020), SIE-1 (Wilson et al., 2021), SPR-1 (Vella et al., 2013), and LRA-8 (Pedroso et al., 2017). When the crystal structures of the eight selected B3 MBLs were superimposed, the RMSD values among the structures were between 0.90 and 1.73 Å, with an average value of 1.51 Å in 231 residues. These values suggest that the overall structures are well conserved within the B3 subclass (Supplementary Table 5). The B3 MBLs showed significant structural differences in the core scaffold compared to B1 and B2 MBLs (Figure 3C). In the central β-sheets of the core scaffold, β1, β2, and β3 from the first β-sheet are very short and a long N-terminal tail provides a flexible conformation of eL1; the second β-sheet consists of five β-strands instead of six β-strands, and the C-terminal β13 is changed to the helix (Supplementary Figure 7). The additional helix α3′ exists immediately after α3 and before β7. The long N-terminal tail forming eL1 showed varied relative positions in the different B3 MBL structures of L1, GOB-1, CSR-1, and AIM-1, which could affect the catalytic activity (Pedroso et al., 2020).

Although two zinc ions are bound in B3 MBLs, their coordination is different from that of B1 MBLs (Pedroso et al., 2020). In canonical B3 MBLs, the Zn1 ion is coordinated with three His residues like B1 MBLs. However, the Zn2 site of B3 MBLs was different from those of both B1 and B2 MBLs. The L3 of B3 MBLs was shorter than those of B1 and B2 MBLs without the Zn2 ion coordinating Cys residue, and its conformation was also different (Figure 4C). A compensatory His residue from the L1 loop is additionally involved to bind the Zn2 ion from the bottom position (Figure 3C). In the Zn1 site, the first His residue from the L1 loop is sometimes replaced with a Gln residue (Supplementary Figure 4). Compared to the corresponding Asn residue of B2 MBLs, the longer Gln side chain in B3 MBLs could be sufficient to coordinate and hold the Zn1 ion. Recently, B3 MBL variants with different zinc coordination residues in both zinc sites were also found, which implies the active site of B3 MBLs appears to be more diverse than those of B1 and B2 MBLs (Pedroso et al., 2020).

eL1, eL2, and eL3 of B3 MBLs were different from those of B1 and B2 MBLs (Figure 3C). In AIM-1, eL1 includes a long N-terminal tail loop, which exists close to the Zn2 site and forms the left wall of the substrate-binding pocket (Figure 3C and Supplementary Figure 7). Although the secondary structures of eL1 are different in B1 and B3 MBLs, the superimposed positions are similar. eL2 has the characteristic additional helix α3′, which is close to the long α3 in B2 members but has a different orientation (Figure 3C). The most significant change occurred in eL3. Without a Zn2-coordinating residue from L3, eL3 stretches straight outward from the second β-sheet, which causes eL3 to shift to the right side and generates a large hole in the upper and left lip. Generally, B3 MBLs have the upper left open space in the substrate-binding pocket to accommodate bulky R groups on β-lactam substrates (Figure 4C and Supplementary Figure 7).

### Comparison Among the Metallo-β-Lactamases of the Three Subclasses

MBLs have zinc ion(s) in the active site on the top of two central β-sheets, and the substrate-binding pocket is formed mainly from the external loops protruding above the canonical αβ/βα MBL scaffold. Structural comparison among the MBLs B1 NDM-1, B2 CphA, and B3 AIM-1 revealed the characteristic structural features of each subclass in the core scaffold, zinc coordination, and substrate-binding pocket.

In the active site, both the Zn1 and Zn2 sites of NDM-1 and CphA were well superimposed (Figure 3). Although CphA does not have the Zn1 ion, the corresponding position of the Zn1 site was well superimposed. However, the zinc binding sites of AIM-1 were shifted to the lower left position compared to those of NDM-1 due to the change in the core scaffold. Within B3 MBLs, the correlation of the metal-metal distance in the active site was observed (Wilson et al., 2021). Interestingly, even in the shifted or different zinc positions, the interatomic distance between the two zinc ions was almost the same as that observed between NDM-1 and AIM-1 (3.45 Å). The average distance in all the selected B1 and B3 MBLs was 3.56 Å (Supplementary Table 6), which is sufficient to bind and coordinate the catalytic water molecule to hydrolyze the β-lactam ring of the substrates in the active site.

The shape of the substrate-binding pocket, which is mainly formed by the core scaffold and external loops, is important for substrate binding according to substrate specificity. Compared to the conserved coordination geometry of zinc binding sites in each subclass and the interatomic distance between the Zn1 and Zn2 ions, the structure of the substrate-binding pocket varies among the three subclasses: B1 MBLs have a long eL1, short eL2, and long eL3; B2 MBLs have a short eL1, long eL2, and long eL3; and B3 MBLs have long eL1, long eL2, and short eL3 (Figures 3, 4).

B1 MBLs have an open space in the left and central bottom positions in the substrate-binding pocket (Figure 4A). B2 MBLs have a narrow open space on the left side horizontal to the Zn1 and Zn2 sites. Furthermore, the bottom is blocked by the long eL2, forming a narrow substrate-binding pocket (Figure 4B). In B3 MBLs, both zinc ions are extensively exposed to solutions, and the left and upper sides of the substrate-binding pocket are wide open (Figure 4C). Only the short eL3 provides a shallow barrier on the upper and right sides of the substrate-binding pocket.

### β-Lactam-Bound Metallo-β-Lactamases

The structures of β-lactam-bound MBLs were superimposed to study substrate recognition in the varied substrate-binding pockets of MBLs: the hydrolyzed product β-lactam-bound MBL structures were used instead of substrate β-lactam-bound MBL structures due to unavailability (Figure 4). The bound β-lactams showed a well-conserved conformation in the active site (Supplementary Figure 8). The cleavable C-N bond of β-lactams was faced toward the zinc site(s) within the distance of direct interactions, in which the β-lactam ring can be easily attacked by a catalytic hydroxide ion bound to zinc ion(s) (Supplementary Figures 1, 8). In the bound structures, the existing carboxyl and carbonyl groups of the core β-lactams were directly bound to the zinc ion(s) in the active sites of MBLs. Accordingly, there is little space to accommodate additional structural motifs in the β-lactam positions.

All B1, B2, and B3 MBLs have open space on the left side between potentially flexible eL1 and eL3; B2 MBLs have a narrow pocket, B1 MBLs have a medium-sized pocket, and B3 MBLs have a wide-open pocket. The left side of the substrate-binding pocket can accommodate the various R groups at the five- or six-membered ring side of the core β-lactam scaffold with a carboxylate (blue shade). The bottom pocket between eL1 and eL2 is noticeably wide only in B1 MBLs and is limited in B2 and B3 MBLs. The bottom side of the substrate-binding pocket binds the R groups on the β-lactam ring side (red shade) and allows only limited structural substitutions.

**FIGURE 5:**
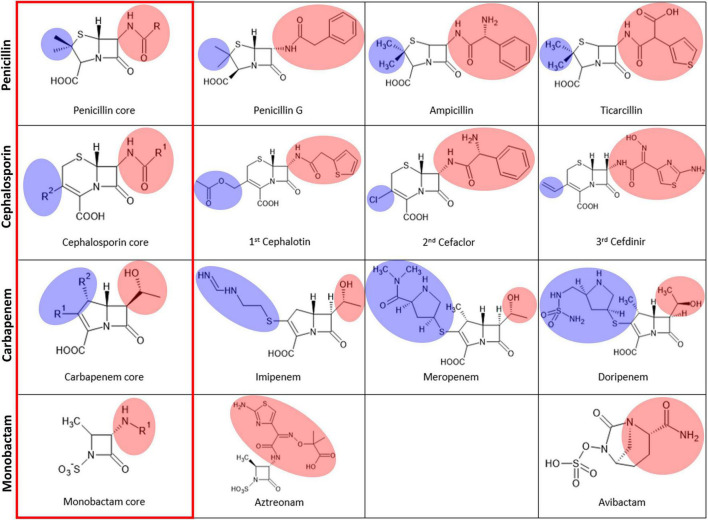
The chemical structures of the four major classes of β-lactams. The left column, labeled with a red rectangle, shows the core scaffold of each type of β-lactam. The substituted R groups at the four-member β-lactam ring are shaded in pink, and those at the five or six-membered dihydropyrrole ring or dihydrothiazine ring next to the β-lactam ring are shaded in blue.

Thus far, various modifications have been introduced in the different R positions in the core β-lactam scaffold for better efficacy in all classes of β-lactams, including penicillins, carbapenems, cephalosporins, and monobactams (Figure 5). Modifications, especially bulky ones, can cause steric hindrance in the varied substrate-binding pockets in B1, B2, and B3 MBLs. Among the five β-lactams in the six substrate-bound structures, penicillin G and cephalosporin have an additional bulky motif at the β-lactam ring side (red shade), and meropenem and biapenem have one at the other five- or six-member ring side (blue shade). This motif is bound to the open space on the bottom (red shade) and left side (blue shade) of the substrate-binding pocket, respectively (Figures 4, 5). The available room on the bottom and left side of the pockets of B1, B2, and B3 MBLs is important for binding a specific β-lactam antibiotic for substrate specificity.

### Inhibitor-Bound Metallo-β-Lactamases

**FIGURE 6:**
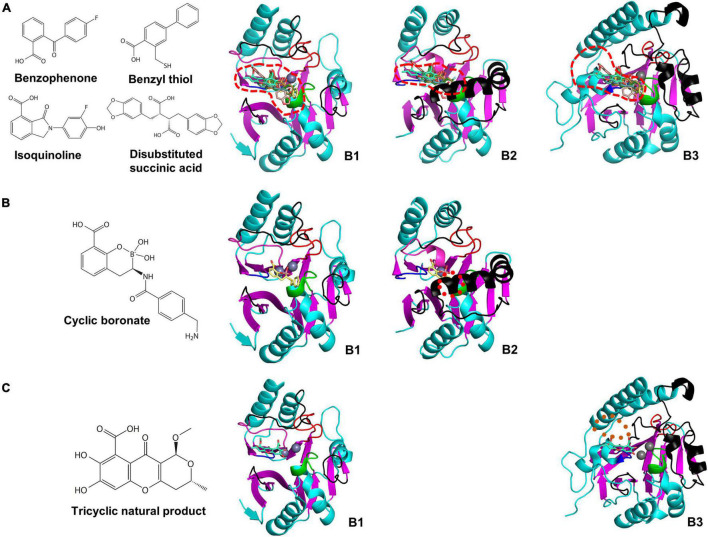
The chemical structures of B1 MBL inhibitors and their superimposed structures on the representative B1, B2, and B3 MBLs. The chemical structures of **(A)** benzophenone, benzyl thiol, isoquinoline, disubstituted succinic acid, **(B)** cyclic boronate, and **(C)** tricyclic natural product, and their superimposed structures on the B1, B2, and B3 MBLs of NDM-1, CphA, and AIM-1, respectively. The substrate-binding pockets of the B1, B2, and B3 MBLs are represented by red dashed lines. The red dotted circle represents the clashed region between the superimposed cyclic boronate and the substrate-binding pocket of B2 MBL. The brown dotted oval represents the space between the tricyclic natural product and substrate-binding pocket of B3 MBL.

**FIGURE 7:**
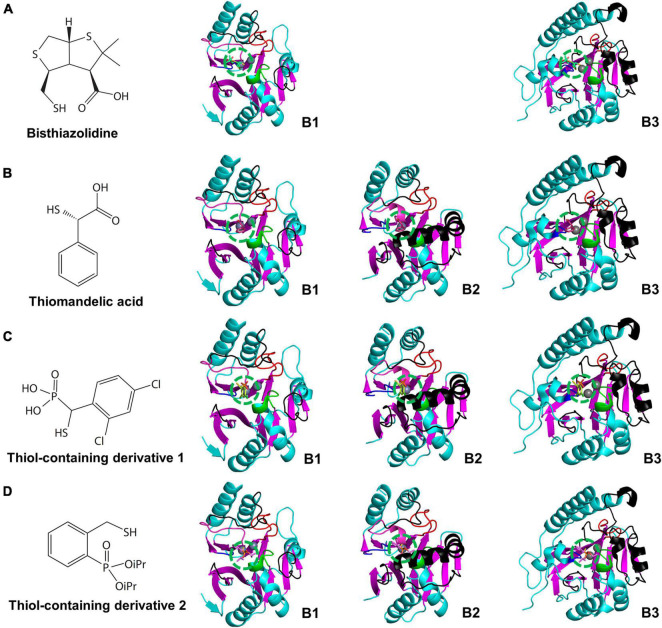
The chemical structures of cross-class MBL inhibitors and their superimposed structures on the representative B1, B2, and B3 MBLs. The chemical structures of **(A)** bisthiazolidine, **(B)** thiomandelic acid, **(C,D)** thiol-containing derivatives and their superimposed structures on the B1, B2, and B3 MBLs of NDM-1, CphA, and AIM-1, respectively. The binding sites of the cross-class inhibitors are represented by green dashed circles. The binding sites are well conserved on top of the zinc-binding site(s). The superimposed cross-class MBL inhibitors with small globular shapes show limited steric hindrances with the substrate-binding pockets of the B1, B2, and B3 MBLs. In (D), iPR represents isopropyl group.

We selected the MBL inhibitors having the co-crystal structure and the inhibitory mechanism of metal ion-binding (Supplementary Table 7; Ju et al., 2018). The inhibitor-bound MBLs were superimposed to study the inhibitory spectrum in the varied substrate-binding pockets. The inhibitors are divided into B1 and cross-class inhibitors, which have inhibitory activity on B1 MBLs (Figure 6) and MBLs of more than a subclass (Figure 7), respectively, based on limited enzyme assay results. The B1 MBL inhibitors include benzophenone (Christopeit et al., 2015), benzyl thiol (Cain et al., 2018), isoquinoline (Li et al., 2017), disubstituted succinic acid (Toney et al., 2001), cyclic boronate (Brem et al., 2016), tricyclic natural product (Payne et al., 2002), and biphenyl tetrazole (Toney et al., 1998) and the cross-class inhibitors, bisthiazolidine (Hinchliffe et al., 2016), thiomandelic acid (Mollard et al., 2001; Karsisiotis et al., 2013), and thiol-containing derivatives (Lassaux et al., 2010). The B1 inhibitors were developed against clinically relevant B1 MBLs, and their inhibitory activities were primarily measured on only B1 MBLs; accordingly, some B1 inhibitors might inhibit other subclass MBLs. Among them, cyclic boronate and tricyclic natural product have selective inhibitory activity on B1 MBLs. Based on the proposed structural characteristics of B1, B2, and B3 subclasses, the structure of cyclic boronate is well fitted within the substrate-binding pocket of B1 MBL, but the steric hindrance is shown with that of B2 MBL (Figure 6B). The structure of the tricyclic natural product is also well fitted in that of B1 MBL, but the loosen interaction is shown within the wide-open substrate-binding pocket of B3 (Figure 6C).

The cross-class MBL inhibitors show relatively smaller and globular shapes rather than the elongated shapes of the B1 inhibitors, which fit well within the center of the substrate-binding pocket on top of the zinc-binding site (Figure 7). The central pocket is conserved and free from the steric hindrance with eL1-3 within MBLs of all subclasses. Significantly, the thiol-containing derivatives showing similarity with the thiomandelic acid were co-crystallized with the B2 subclass CphA, which has the narrow substrate-binding pocket. The thiol-containing derivatives showed comparable inhibitory effects on MBLs of all three subclasses (Supplementary Table 7). In addition to the inhibitors co-crystallized with MBLs, potent MBL inhibitors having trifluoromethyl ketones and alcohols, dicarboxylic acids, thiols, sulfates, hydroxamates, tetrazoles, and sulfonamides as scaffolds have been studied with molecular modeling and docking methods (McGeary et al., 2014, 2017; Arjomandi et al., 2016; Yusof et al., 2016).

## Discussion

The varied substrate-binding pockets of B1, B2, and B3 MBLs makes it difficult to develop a broad-spectrum inhibitor against all subclasses of MBLs. However, the zinc sites are relatively well conserved in all MBLs; the relative distance between two zinc ions is almost the same in all MBLs, except for the loss of Zn1 in the B2 subclass. Considering the conserved zinc sites and the opposingly varied substrate-binding pockets of MBLs, the catalytic value of *k*_cat_ could be affected mainly by the catalytic hydroxide ion bound at the zinc ion(s). The K_m_ value for the affinity for the substrate could be more affected by the substrate-binding pockets formed by the external loops.

From the systematic structure analysis of all MBLs, a strategy to develop a broad-spectrum inhibitor could involve targeting a metal-binding inhibitor to the zinc ion(s) in the active site. This could involve an inhibitor that had a sufficiently small size or flexible structure to fit into the diverse substrate-binding pockets of all subclasses of MBLs. Aspergillomarasmine A might have a similar working mechanism, as it has a flexible scaffold with metal chelator activity and successfully inhibits MBLs (King et al., 2014; Mojica et al., 2021); however, its clinical efficacy remains to be determined. Zinc ions are abundant in living organisms. Approximately 1,600 proteins have been proposed as zinc proteins in human, and these proteins have catalytic and structural roles (Andreini et al., 2006). The human zinc-binding proteins are potential off-targets, and the resulting side effects should be considered. It is necessary to identify the window span for inhibitors with a high affinity for many MBLs and a low affinity for off-targets in humans.

The structural comparison among the selected MBLs of the three subclasses and the β-lactam-bound structures demonstrates the conserved features and unique characteristics of each subclass. The proposed unique characteristics of the substrate-binding pocket in B1, B2, and B3 MBLs were further verified with narrow and broad-spectrum MBL inhibitors. The cross-class inhibitors are found to bind to the central substrate-binding pocket, which is commonly available in all subclasses, with the complementary chemical structures.

Different from traditional MBLs, there are also non-canonical MBLs such as SPS-1 (Cheng et al., 2018) and SPM-1 (Brem et al., 2015) that belong to the B1 and B3 subclasses, respectively. SPS-1 has a long eL2 (Supplementary Figure 2), which showed a long bent α3 helix forming eL2 similar to that of the B2 members (Supplementary Figure 9A). SPM-1 showed two different open and close conformations in the α3 (Supplementary Figure 9B). These findings imply that despite decades of β-lactam-related research by international groups, the current classification and structural information of B1, B2, and B3 MBLs could be still incomplete and limited. Even the directed evolution study of AIM-1 showed the substrate preference relevant amino acids are not necessarily near the catalytic center of the enzyme (Hou et al., 2017). Cautions should be exerted when making a conclusion related to MBLs based on the currently available structural information.

This study systematically compared MBLs of all three subclasses altogether in sequence, structure, and substrate specificity. The MBL structures are scrutinized in the core scaffold, zinc-coordination loops of L1-4, and substrate-binding pocket-forming external loops of eL1-3 for the structure-function relationships in terms of substrate specificity. Because all MBLs have the common comprising moieties, the sequences and structures of characteristic moieties could be compared simultaneously among multiple MBLs. The multiple comparative statistics in the sequence identities and RMSD values among MBLs are used to verify the conservation and difference among MBLs of the same and different subclasses. The characteristic structural differences are used to explain the substrate specificity of MBLs. However, the currently available structural information of MBLs is limited. For example, there is no structure of any unbroken substrate β-lactam-bound MBL and only hydrolyzed product β-lactam-bound MBLs are available. There are many MBLs and variants with uncharacterized activities on substrates and unknown structures, making it hard to generalize the current understandings as the canonical structural features and substrate specificity of classified MBLs. Consequently, the systematic comparative study of several tens of multiple MBLs in the sequence, structure, and structure-function relationships is still limited, but could be used as a valuable platform to understand and predict the mechanism and substrate specificity of existing or newly found MBLs. The structural insights of MBLs are also valuable to develop a broad-spectrum inhibitor against MBLs.

## Data Availability Statement

The datasets presented in this study can be found in online repositories. The names of the repository/repositories and accession number(s) can be found in the article/Supplementary Material.

## Author Contributions

YY, SH, YSP, HP, DK, YSK, YDK, SK, JHL, JHJ, SHL, and L-WK: investigation and writing. YY, SH, YSP, and L-WK: methodology. JHL, JHJ, SHL, and L-WK: funding acquisition. All authors have read and agreed to the published version of the manuscript.

## Conflict of Interest

The authors declare that the research was conducted in the absence of any commercial or financial relationships that could be construed as a potential conflict of interest.

## Publisher’s Note

All claims expressed in this article are solely those of the authors and do not necessarily represent those of their affiliated organizations, or those of the publisher, the editors and the reviewers. Any product that may be evaluated in this article, or claim that may be made by its manufacturer, is not guaranteed or endorsed by the publisher.
