# Supplementary material for: Structural Insights for Core Scaffold and Substrate Specificity of B1, B2, and B3 Metallo-β-Lactamases
Source: Front Microbiol. 2022 Jan 13;12:752535. doi: 10.3389/fmicb.2021.752535 (PMC8792953; doi:10.3389/fmicb.2021.752535)
Supplement: Supplementary file 1 [file Data_Sheet_1.docx]

Supplementary Material

# Supplementary Figures


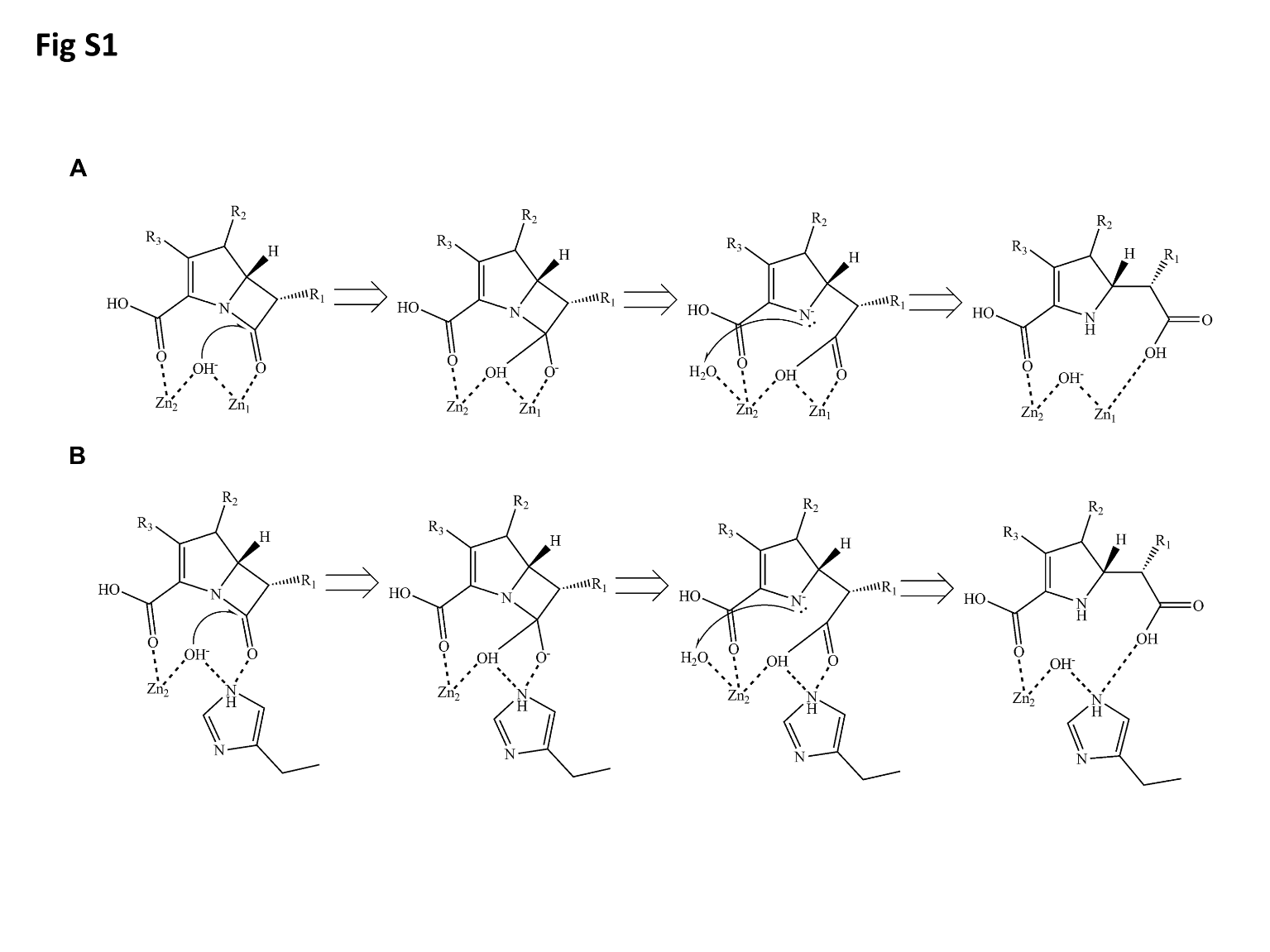


**Figure S1.** The generally proposed catalytic mechanism of metallo-β-lactamases (MBLs). (A) The catalytic mechanism of di-zinc MBLs is used by canonical B1 and B3 MBLs. The catalytic hydroxide ion bridging two zinc ions attacks the carbonyl group and cleaves the C-N bond in the β-lactam ring, and the resulting nitrogen atom is protonated by a nearby acid. (B) The catalytic mechanism of mono-zinc MBLs is used by canonical B2 MBLs. The catalytic hydroxide ion bound between Zn2 and a His residue, instead of between two zinc ions, plays a catalytic role.


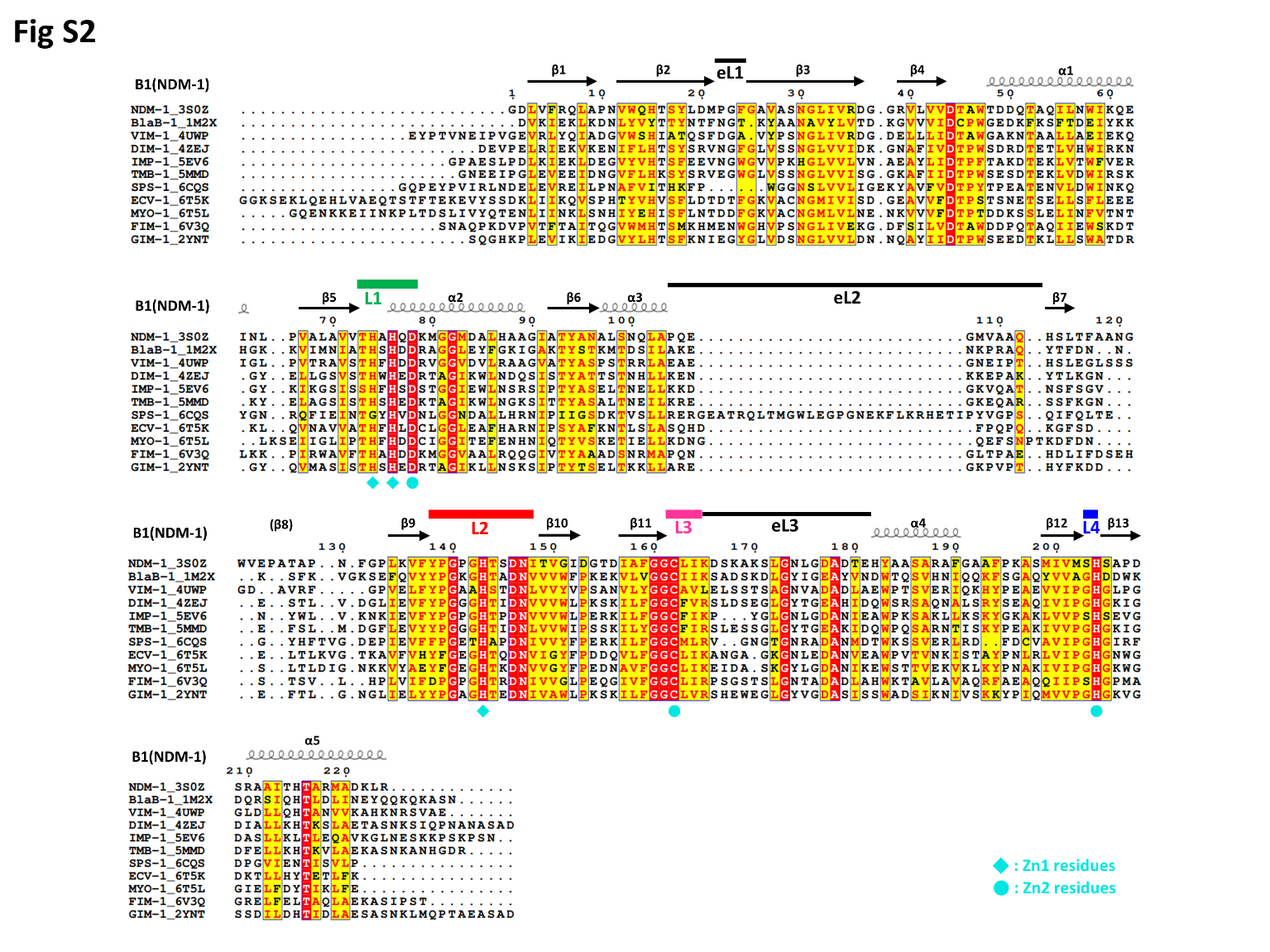


**Figure S2.** The structural sequence alignment of B1 metallo-β-lactamases (MBLs). The secondary structures of NDM-1 are shown at the top; the secondary structures are labeled based on the core scaffold of thirteen β-strands and five α-helices. The zinc-coordinating residues of B1 MBLs are shown as cyan diamonds and circles. The four zinc-coordinating loops, L1, L2, L3, and L4, are shown as thick green, red, pink, and blue lines, respectively. The three external loops, eL1, eL2, and eL3, are shown as thin black lines. The missing β8 (parenthesized) exists as a helix in NDM-1, as mentioned in Figure 1A.


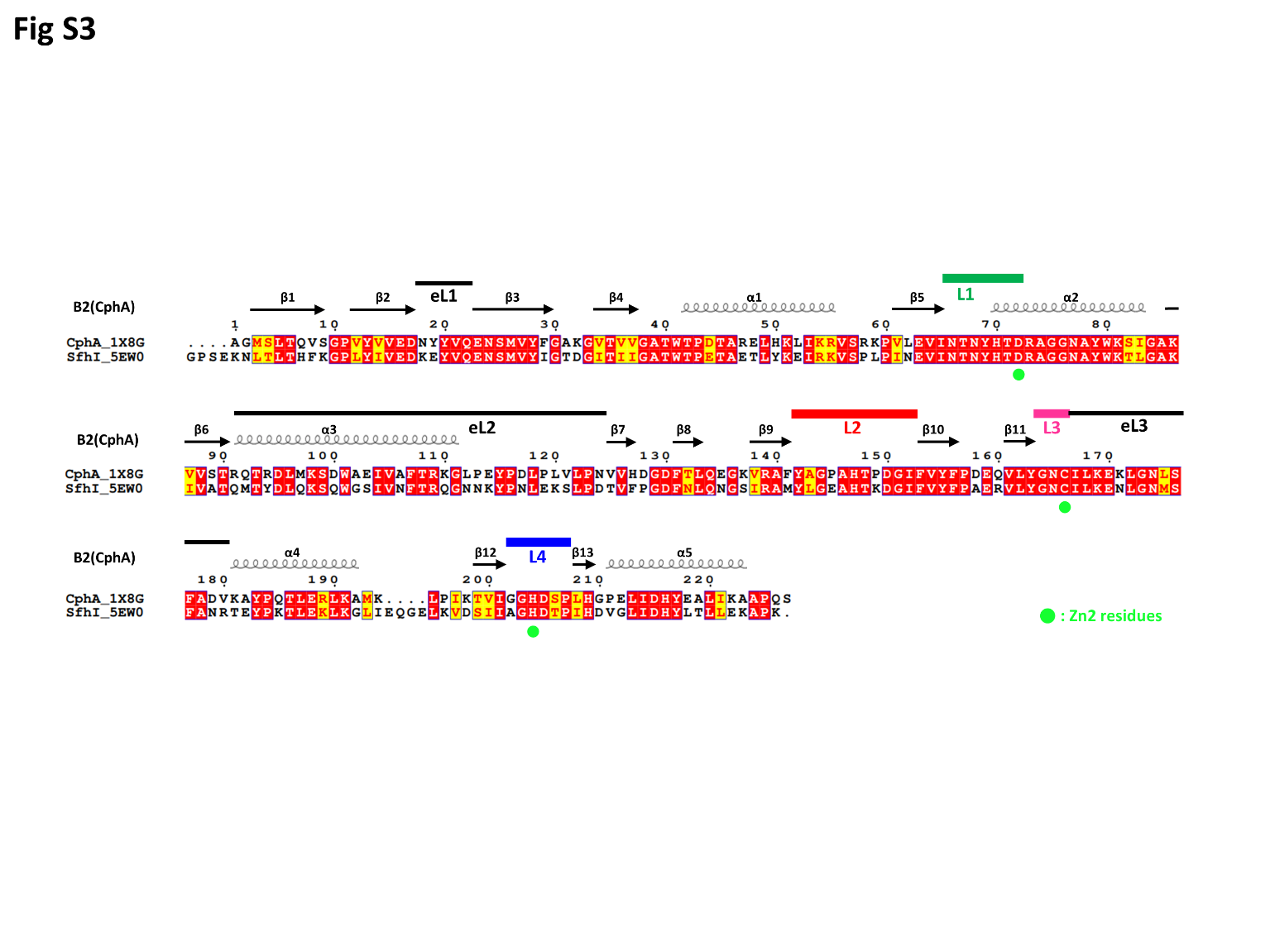


**Figure S3.** The structural sequence alignment of B2 metallo-β-lactamases (MBLs). The secondary structures of CphA are shown at the top; the secondary structures are labeled based on the core scaffold of 13 β-strands and 5 α-helices. The zinc-coordinating residues of B2 MBLs are shown as green circles. The four zinc-coordinating loops, L1, L2, L3, and L4, are shown as thick green, red, pink, and blue lines, respectively. The three external loops, eL1, eL2, and eL3, are shown as thin black lines. The helix α3 forming eL2 is long and bent in the middle and blocks the bottom of the substrate-binding pocket, as shown in Figure 4B.


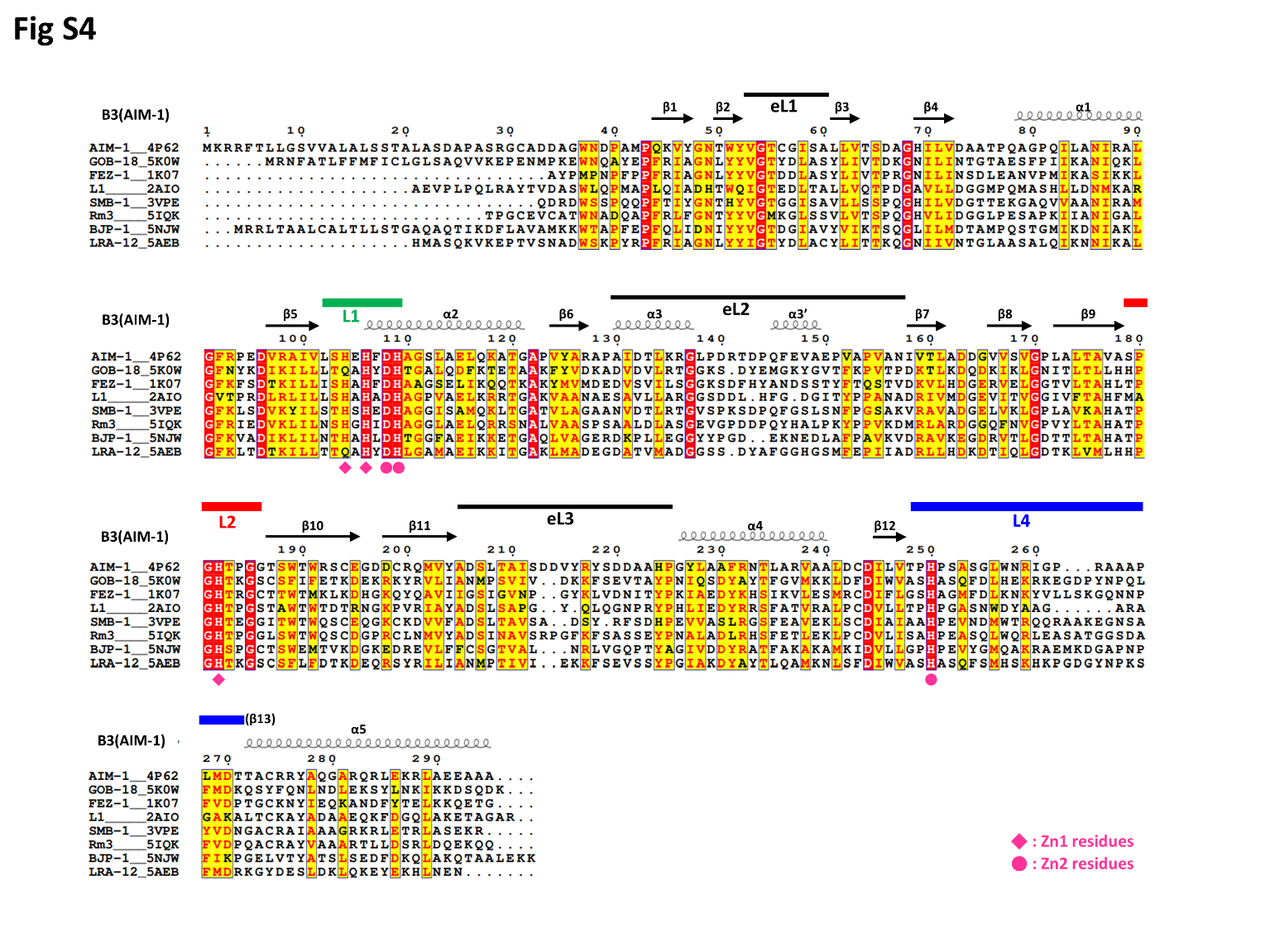


**Figure S4.** The structural sequence alignment of B3 metallo-β-lactamases (MBLs). The secondary structures of AIM-1 are shown at the top; the secondary structures are labeled based on the core scaffold of thirteen β-strands and five α-helices. The zinc-coordinating residues of B3 MBLs are shown as pink diamonds and circles. The four zinc-coordinating loops L1, L2, and L4 are shown as thick green, red, and blue lines, respectively. L3 is missing in B3 MBLs because there is no Zn2-coordinating residue from L3. The three external loops, eL1, eL2, and eL3, are shown as thin black lines. There is an additional helix of α3′ or loop after α3, which forms the bottom and right sides of the substrate-binding pocket in canonical B3 MBLs, as shown in Figure 4C. There is a long N-terminal loop before β1, which mainly forms eL1 by compensating for the short β1–3 compared to those of B1 and B2 MBLs. In canonical B3 MBLs, β13 (parenthesized), which is close to L4, is changed to a helix.


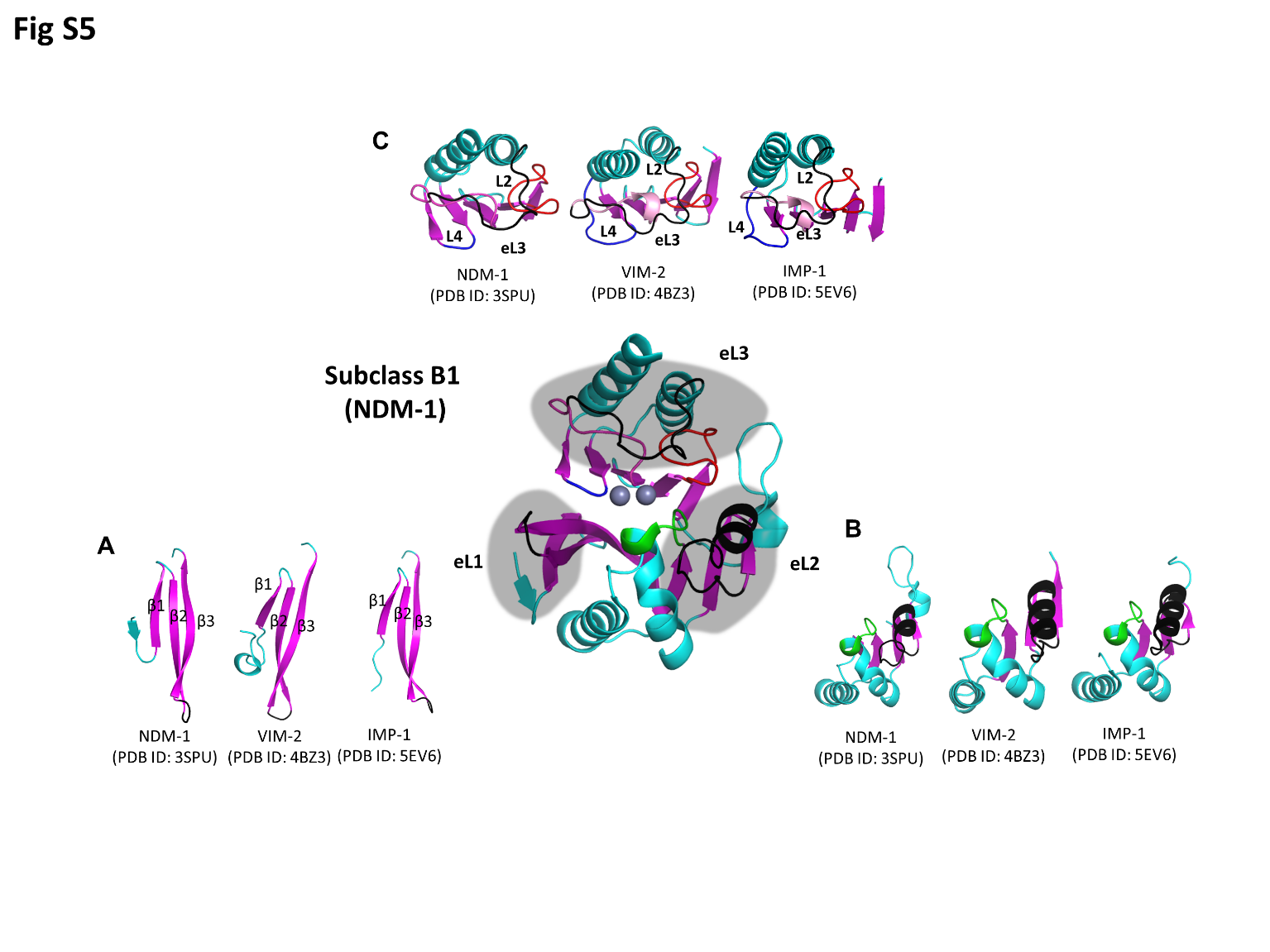


**Figure S5.** The structure of the varied external loops of B1 metallo-β-lactamases (MBLs). The crystal structure of NDM-1, the representative B1 MBL in this study, is shown in the middle, with the grey shading indicating (A) eL1, (B) eL2, and (C) eL3. The varied structures of eL1, eL2, and eL3 of B1 MBLs are shown in parallel. In eL1, β2 and β3 are approximately two times longer than β1 and have a flexible conformation. The eL2 including α3 is much shorter than those from B2 and B3 MBLs, which generates a moderate space at the bottom of the substrate-binding pocket. The substituted R groups at the β-lactam ring bound to the bottom, as shown in Figure 4A. eL3, including Zn2-coordinating L3, constitutes the entire upper lip of the substrate-binding pocket and is surrounded from outside by two long α4 and α5 helices of the core scaffold.


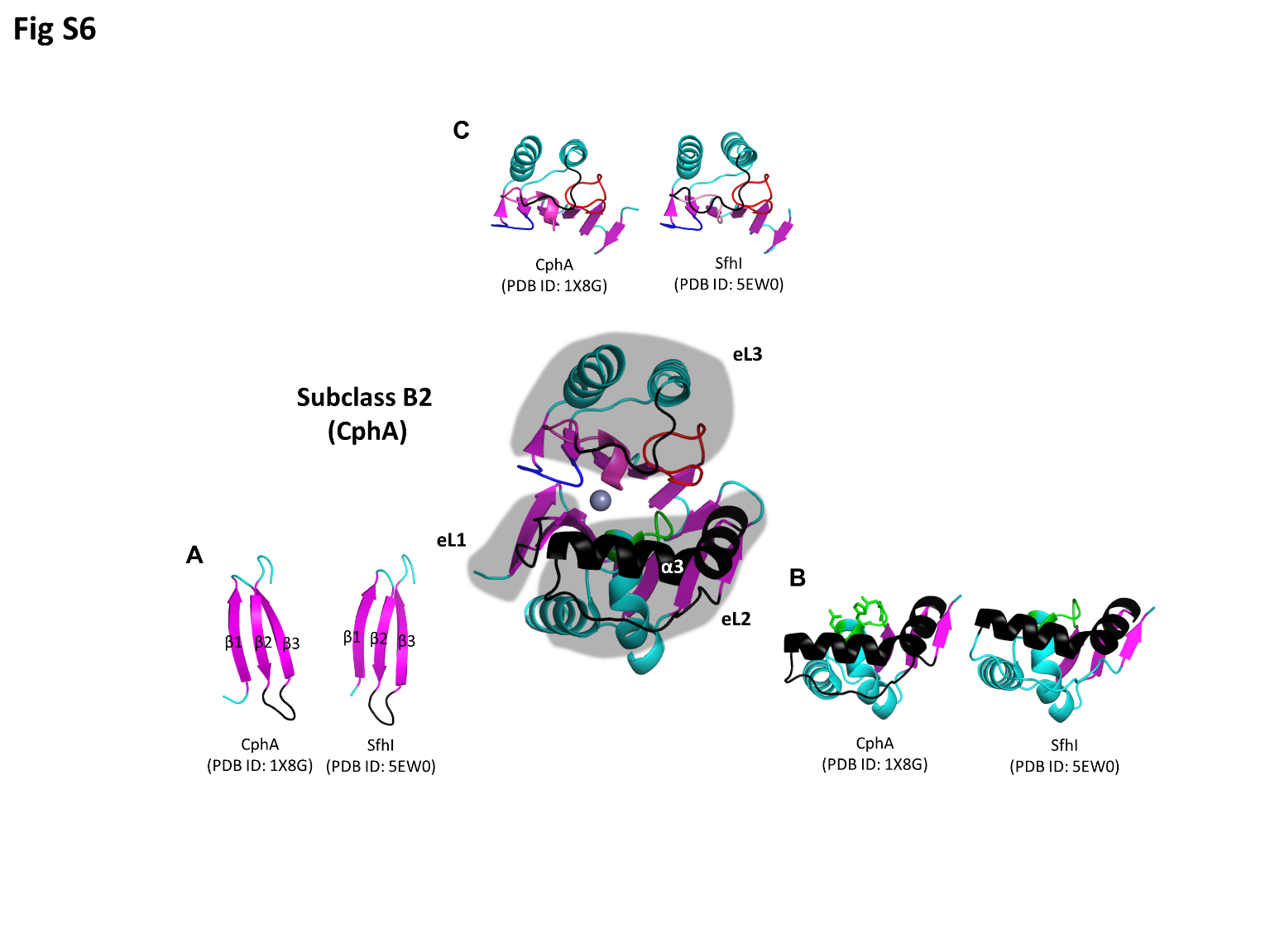


**Figure S6.** The structure of the varied external loops of B2 metallo-β-lactamases (MBLs). The crystal structure of CphA, the representative B2 MBL in this study, is shown in the middle, with the grey shading indicating (A) eL1, (B) eL2, and (C) eL3. The varied structures of eL1, eL2, and eL3 of B2 MBLs are shown in parallel. In eL1, β2 and β3 are almost the same size as the short β1. Accordingly, the tips of β2 and β3 have a flexible loop conformation. The eL2 forming α3 is much longer and bent compared to that of B1 and B3 MBLs, which completely closes the open space at the bottom of the substrate-binding pocket and forms a narrow substrate-binding pocket. eL3, including Zn2-coordinating L3, has a slightly shorter length compared to that of B1 MBLs, but has a similar conformation to that of B1 MBLs. It constitutes the entire upper lip of the substrate-binding pocket and is surrounded from the outside by two long α4 and α5 helices of the core scaffold.


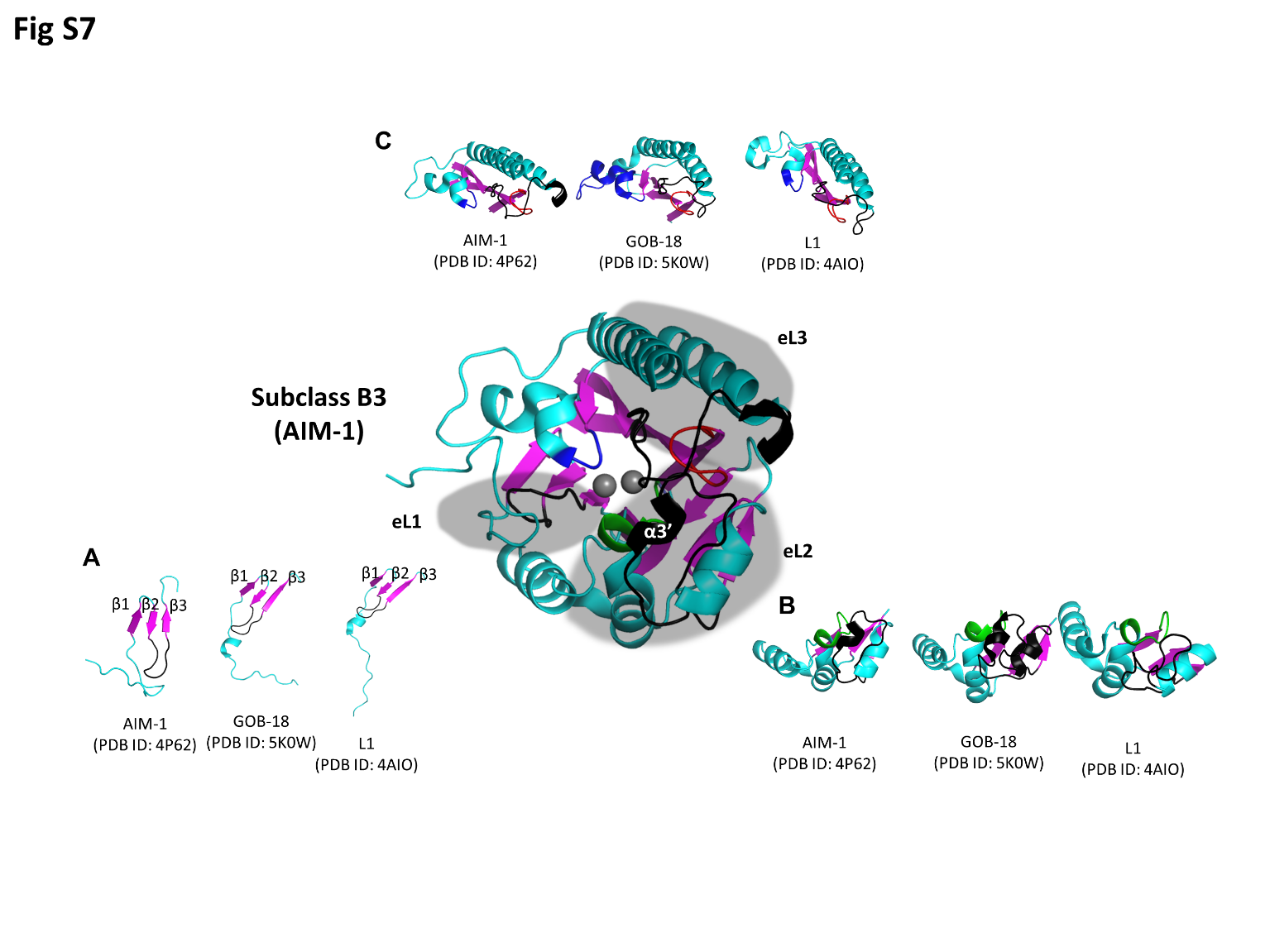


**Figure S7.** The structure of the varied external loops of B3 metallo-β-lactamases (MBLs). The crystal structure of AIM-1, the representative B3 MBL in this study, is shown in the middle, with grey shading indicating (A) eL1, (B) eL2, and (C) eL3. The varied structures of eL1, eL2, and eL3 of B3 MBLs are shown in parallel. In eL1, the length of β1–3 is the shortest among all the three subclasses. The N-terminal loop before β1 is especially long and forms a major part of eL1. The eL2 including α3 is long and often has an additional helix α3′ or loop, which forms the bottom and right walls in the substrate-binding pocket. eL3, which is missing the Zn2-coordinating L3, shows the shifted conformation of the right side compared to that of B1 and B2 MBLs. This shift causes the wide and open structure in the upper left lip of the substrate-binding pocket. In canonical B3 MBLs, β13 (parenthesized), which is close to L4, is changed to a helix.


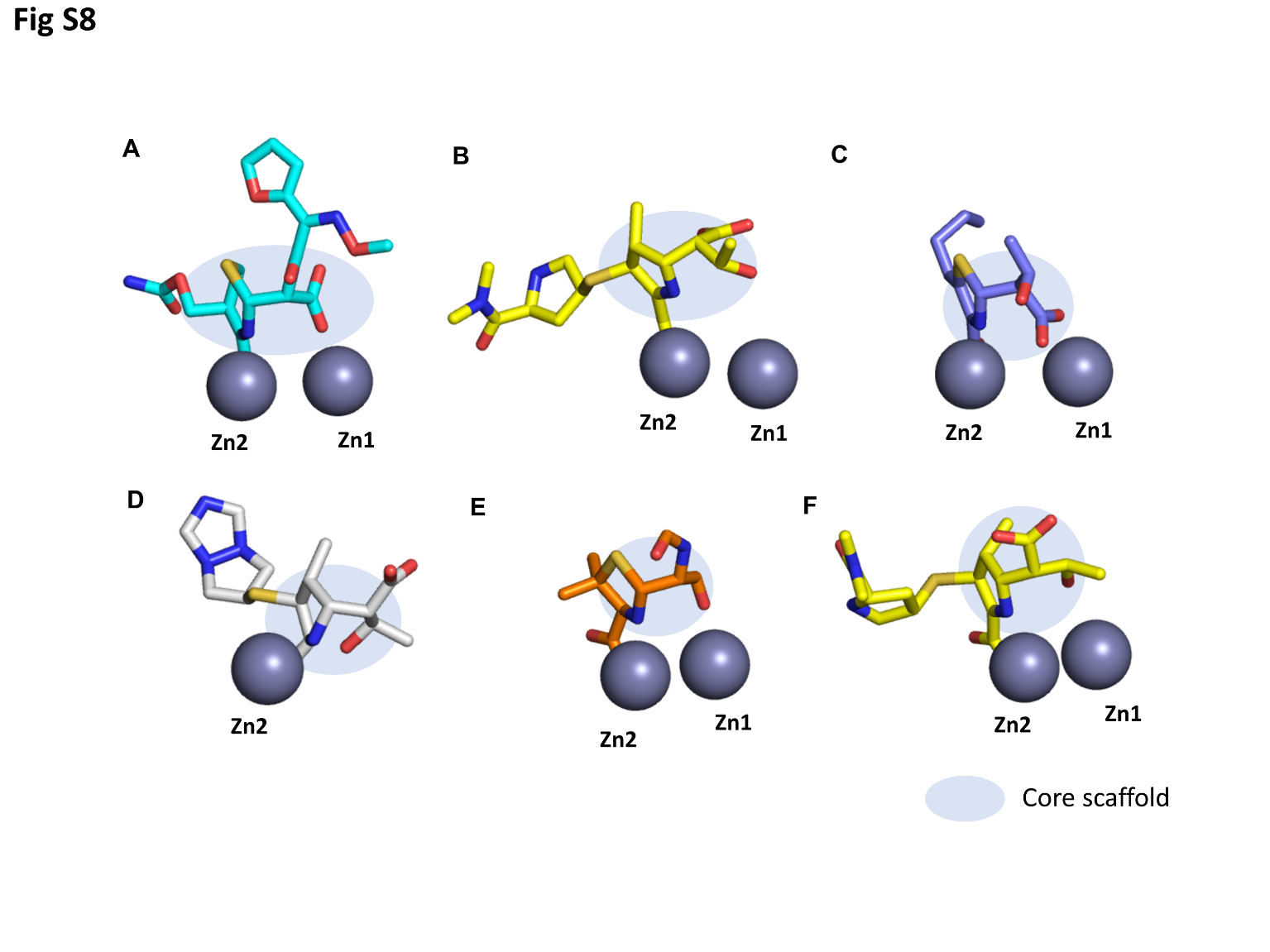


**Figure S8.** The conserved bound conformations of the hydrolyzed β-lactams in metallo-β-lactamases (MBLs). (A) Cephalosporin bound to NDM-1 of the B1 subclass (PDB ID: 4RL0) (B) Meropenem bound to VIM-1 of the B1 subclass (PDB ID: 5N5I) (C) Faropenem bound to VIM-2 of the B1 subclass (PDB ID: 7A5Z) (D) Biapenem bound to CphA of the B2 subclass (PDB ID: 1X8I) (E) Penicillin G bound to L1 of the B3 subclass (PDB ID: 6U0Z) (F) Meropenem bound to SMB-1 of the B3 subclass (PDB ID: 5AXO). All the determined structures show the hydrolyzed forms of the β-lactams. The core scaffold with the cleaved β-lactam ring and the next five or six-member ring is shaded in grey.


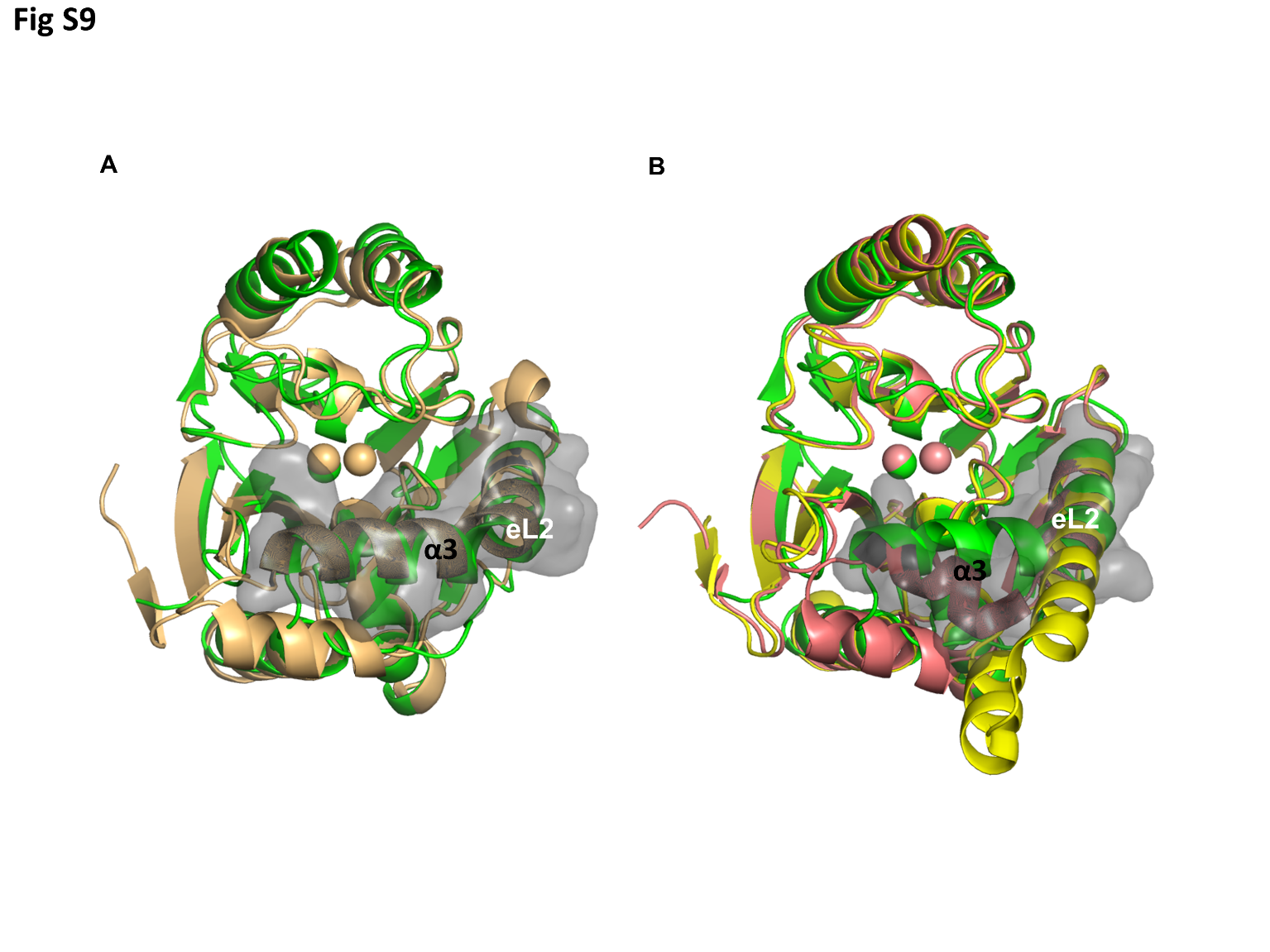


**Figure S9.** The superimposed structures of SPS-1 and SPM-1 on CphA. (A) The superimposed structures of SPS-1 (pale yellow, PDB ID: 6CQS) and CphA (green, 1X8G). The surface of the bent α3 helix forming eL2 of SPS-1 is shown in grey. (B) The superimposed structures of the open (yellow, 2FHX) and closed (dark salmon, 4BP0) forms of SPM-1 and CphA (green, 1X8G). The surface of the α3 helix forming eL2 (close conformation) of SPM-1 is shown in grey.

# Supplementary Tables

**Table S1.** The sequence identity among B1, B2, and B3 metallo-β-lactamases (MBLs)

| Protein | Percentage sequence identity | | | | | | | | | | | | | | | | | | |  | |  |
| --- | --- | --- | --- | --- | --- | --- | --- | --- | --- | --- | --- | --- | --- | --- | --- | --- | --- | --- | --- | --- | --- | --- |
|  | NDM-1 | BlaB-1 | VIM-2 | DIM-1 | IMP-1 | TMB-1 | SPS-1 | ECV-1 | MYO-1 | FIM-1 | GIM-1 | CphA | Sfh1 | AIM-1 | GOB-18 | FEZ-1 | Rm3 | SMB-1 | L1 | | BJP-1 | LRA-12 |
| NDM-1 | 100 | 25.38 | 38.69 | 32.14 | 34.20 | 35.20 | 22.68 | 31.09 | 25 | 47.24 | 30.61 | 23.16 | 20.00 | 16.94 | 11.89 | 12.97 | 12.90 | 16.94 | 18.86 | | 17.58 | 17.30 |
| BlaB-1 | 25.38 | 100 | 25.63 | 32.10 | 30.83 | 30.17 | 24.67 | 29.82 | 28.45 | 19.01 | 32.23 | 26.79 | 25.79 | 13.64 | 19.73 | 17.73 | 17.18 | 17.84 | 14.95 | | 16.22 | 14.54 |
| VIM-2 | 38.69 | 25.63 | 100 | 31.36 | 30.90 | 31.78 | 25.74 | 29.88 | 27.53 | 35.57 | 32.77 | 25.11 | 23.45 | 14.71 | 10.34 | 12.95 | 16.05 | 13.27 | 16.45 | | 17.24 | 9.83 |
| DIM-1 | 32.14 | 32.10 | 31.36 | 100 | 45.53 | 62.86 | 29.91 | 28.51 | 31.90 | 31.25 | 51.60 | 20.54 | 22.17 | 11.89 | 11.30 | 11.45 | 11.64 | 13.64 | 14.48 | | 15.28 | 13.42 |
| IMP-1 | 34.20 | 30.83 | 30.90 | 45.53 | 100 | 48.76 | 29.73 | 34.96 | 34.78 | 30.38 | 44.90 | 23.66 | 23.08 | 14.41 | 15.11 | 15.32 | 16.30 | 13.49 | 18.06 | | 17.33 | 13.72 |
| TMB-1 | 35.20 | 30.17 | 31.78 | 62.86 | 48.76 | 100 | 27.80 | 27.75 | 31.47 | 32.22 | 52.05 | 20.18 | 22.27 | 12.61 | 13.78 | 12.61 | 11.95 | 13.02 | 14.81 | | 12.05 | 11.56 |
| SPS-1 | 22.68 | 24.67 | 25.74 | 29.91 | 29.73 | 27.80 | 100 | 29.61 | 25.32 | 22.36 | 29.15 | 22.22 | 24.90 | 14.77 | 17.67 | 11.11 | 16.87 | 16.07 | 16.59 | | 16.31 | 13.81 |
| ECV-1 | 31.09 | 29.82 | 29.88 | 28.51 | 34.96 | 27.75 | 29.61 | 100 | 45.49 | 23.97 | 29.96 | 26.46 | 23.98 | 16.54 | 12.79 | 10.75 | 15.35 | 16.90 | 18.98 | | 14.61 | 10.45 |
| MYO-1 | 25 | 28.45 | 27.53 | 31.90 | 34.78 | 31.47 | 25.32 | 45.49 | 100 | 28.86 | 32.03 | 23.79 | 24.89 | 13.54 | 14.73 | 12.96 | 14.66 | 12.50 | 13.57 | | 14.73 | 15.18 |
| FIM-1 | 47.24 | 19.01 | 35.57 | 31.25 | 30.38 | 32.22 | 22.36 | 23.97 | 28.86 | 100 | 28.75 | 17.83 | 17.78 | 19.83 | 10.96 | 16.29 | 13.81 | 14.93 | 18.22 | | 16.23 | 12.50 |
| GIM-1 | 30.61 | 32.23 | 32.77 | 51.60 | 44.90 | 52.05 | 29.15 | 29.96 | 32.03 | 28.75 | 100 | 21.08 | 30.91 | 15.49 | 13.10 | 14.16 | 14.29 | 15.98 | 20.45 | | 16.67 | 13.91 |
| CphA | 23.16 | 26.79 | 25.11 | 20.54 | 23.66 | 20.18 | 22.22 | 26.46 | 23.79 | 17.83 | 21.08 | 100 | 55.02 | 19.31 | 17.24 | 16.89 | 18.07 | 18.55 | 17.70 | | 20.69 | 18.03 |
| Sfh1 | 20.00 | 25.79 | 23.45 | 22.17 | 23.08 | 22.27 | 24.90 | 23.98 | 24.89 | 17.78 | 20.91 | 55.02 | 100 | 13.60 | 20.18 | 16.74 | 15.88 | 16.67 | 13.57 | | 17.62 | 18.78 |
| AIM-1 | 16.94 | 13.64 | 14.71 | 11.89 | 14.41 | 12.61 | 14.77 | 16.52 | 13.54 | 19.83 | 15.49 | 19.31 | 13.60 | 100 | 25.00 | 23.91 | 42.03 | 44.40 | 35.76 | | 30.31 | 25.36 |
| GOB-18 | 11.89 | 19.73 | 10.34 | 11.30 | 15.11 | 13.78 | 17.67 | 12.79 | 14.73 | 10.96 | 13.10 | 17.24 | 20.18 | 25.00 | 100 | 36.30 | 26.48 | 24.63 | 21.79 | | 31.36 | 54.55 |
| FEZ-1 | 12.97 | 17.73 | 12.95 | 11.45 | 15.32 | 12.61 | 11.11 | 10.75 | 12.96 | 16.29 | 14.16 | 16.89 | 16.74 | 23.91 | 36.30 | 100 | 28.93 | 29.74 | 27.94 | | 35.48 | 34.89 |
| Rm3 | 12.90 | 17.18 | 16.05 | 11.64 | 16.30 | 11.95 | 16.87 | 15.35 | 14.66 | 13.81 | 14.29 | 18.07 | 15.88 | 42.03 | 26.48 | 28.93 | 100 | 43.57 | 36.81 | | 29.97 | 25.78 |
| SMB-1 | 16.94 | 17.84 | 13.27 | 13.64 | 13.49 | 13.02 | 16.07 | 16.90 | 12.50 | 14.93 | 15.98 | 18.55 | 16.67 | 44.40 | 24.63 | 29.74 | 43.57 | 100 | 31.60 | | 29.85 | 27.07 |
| L1 | 18.86 | 14.95 | 16.45 | 14.48 | 18.06 | 14.81 | 16.59 | 18.98 | 13.57 | 18.22 | 20.45 | 17.70 | 13.57 | 35.76 | 21.79 | 27.94 | 36.81 | 31.60 | 100 | | 31.67 | 24.64 |
| BJP-1 | 17.58 | 16.22 | 17.24 | 15.28 | 17.33 | 12.05 | 16.31 | 14.61 | 14.73 | 16.23 | 16.67 | 20.69 | 17.62 | 30.31 | 31.36 | 35.48 | 29.97 | 29.85 | 31.67 | | 100 | 30.39 |
| LRA-12 | 17.30 | 14.54 | 9.83 | 13.42 | 13.72 | 11.56 | 13.81 | 10.45 | 15.18 | 12.50 | 13.91 | 18.03 | 18.78 | 25.36 | 54.55 | 34.89 | 25.78 | 27.07 | 24.64 | | 30.39 | 100 |

The amino acid sequences of the selected MBLs were obtained from the NCBI website. The selected genes were as follows: NDM-1 (from *Klebsiella pneumoniae*, protein ID: AQT38377.1), BlaB-1 (from *Elizabethkingia meningoseptica*, protein ID: SQG05762.1), VIM-2 (from *Pseudomonas aeruginosa*, protein ID: ACH43053.1), DIM-1 (from *Pseudomonas aeruginosa*, protein ID: QFC18500.1), IMP-1 (from *Pseudomonas aeruginosa*, protein ID: BAD14385.1), TMB-1 (from *Enterobacter hormaechei subsp. steigerwaltii*, protein ID: PXY62695.1), SPS-1 (from *Sediminispirochaeta smaragdinae*, protein ID: WP_013255389.1), ECV-1 (from *Echinicola vietnamensis*, protein ID: WP_015266427.1), MYO-1 (from *Myroides odoratimimus*, protein ID: WP_081048762.1), FIM-1 (from *Pseudomonas aeruginosa*, protein ID: AFV91534.1), GIM-1 (from *Pseudomonas aeruginosa*, protein ID: AEX25999.1), CphA (from *Aeromonas hydrophila*, protein ID: QIO20011.1), Sfh1 (from *Serratia fonticola*, protein ID: WP_071766619.1), AIM-1 (from *Pseudomonas aeruginosa*, protein ID: CAQ53840.1), GOB-18 (from *Elizabethkingia meningoseptica*, protein ID: WP_063860560.1), FEZ-1 (from *Fluoribacter gormanii*, protein ID: WP_058468956.1), Rm3 (from *uncultured bacterium*, protein ID: WP_071766622.1), SMB-1 (from *Serratia marcescens*, protein ID: BAL14456.1), L1 (from *Stenotrophomonas maltophilia*, protein ID: ABO60995.1), BJP-1 (from *Bradyrhizobium cosmicum*, protein ID: BAL75272.1), and LRA-12 (from *uncultured bacterium*, protein ID: ACH58990.1).

**Table S2.** The RMSD values between NDM-1 and other B1, B2, and B3 metallo-β-lactamases (MBLs)

| Protein | RMSD values | | | | | | | | | | | | | | | | | | |  | | |  | |
| --- | --- | --- | --- | --- | --- | --- | --- | --- | --- | --- | --- | --- | --- | --- | --- | --- | --- | --- | --- | --- | --- | --- | --- | --- |
|  | NDM-1 | BlaB-1 | VIM-2 | DIM-1 | IMP-1 | TMB-1 | SPS-1 | ECV-1 | MYO-1 | FIM-1 | GIM-1 | CphA | Sfh1 | AIM-1 | GOB-18 | FEZ-1 | Rm3 | SMB-1 | L1 | | BJP-1 | LRA-12 | |  |
| NDM-1 | 0 | 1.7296 | 1.0632 | 1.4502 | 1.4914 | 1.4886 | 1.4718 | 1.4795 | 1.5583 | 1.1838 | 1.525 | 1.4755 | 1.3594 | 2.1905 | 2.3727 | 2.1865 | 2.2689 | 2.229 | 1.9955 | | 2.3407 | 2.4272 | |  |

The PDB IDs of the selected MBLs were as follows: NDM-1 (3S0Z), BlaB-1 (1M2X), VIM-2 (4NQ2), DIM-1 (4ZEJ), IMP-1 (5EV6), TMB-1 (5MMD), SPS-1 (6CQS), ECV-1 (6T5K), MYO-1 (6T5L), FIM-1 (6V3Q), GIM-1 (2YNT), CphA (1X8G), SfhI (5EW0), AIM-1 (4P62), GOB-18 (5K0W), FEZ-1 (1K07), Rm3 (5IQK), SMB-1 (3VPE), L1 (2AIO), BJP-1 (5NJW), and LRA-12 (5AEB).

**Table S3.** The RMSD values between NDM-1 and other B1 metallo-β-lactamases (MBLs)

| Protein | RMSD values (B1-B1) | | | | | | | | | | |
| --- | --- | --- | --- | --- | --- | --- | --- | --- | --- | --- | --- |
|  | NDM-1 | BlaB-1 | VIM-2 | DIM-1 | IMP-1 | TMB-1 | SPS-1 | ECV-1 | MYO-1 | FIM-1 | GIM-1 |
| NDM-1 | 0 | 1.7565 | 1.0632 | 1.4502 | 1.4914 | 1.4886 | 1.4718 | 1.4795 | 1.5583 | 1.1838 | 1.525 |

The PDB IDs of the selected MBLs were as follows: NDM-1 (3S0Z), BlaB-1 (1M2X), VIM-2 (4NQ2), DIM-1 (4ZEJ), IMP-1 (5EV6), TMB-1 (5MMD), SPS-1 (6CQS), ECV-1 (6T5K), MYO-1 (6T5L), FIM-1 (6V3Q), and GIM-1 (2YNT).

**Table S4.** The RMSD values between CphA and the other B2 subclass member of SfhI

| Protein | RMSD values (B2-B2) | |
| --- | --- | --- |
|  | CphA | Sfh1 |
| CphA | 0 | 0.6487 |

The PDB IDs of selected MBLs are as following: CphA (1X8G) and SfhI (5EW0).

**Table S5.** The RMSD values between AIM-1 and other B3 metallo-β-lactamases (MBLs)

| Protein | RMSD values (B3-B3) | | | | | | | | |
| --- | --- | --- | --- | --- | --- | --- | --- | --- | --- |
|  | AIM-1 | GOB-18 | FEZ-1 | Rm3 | SMB-1 | L1 | BJP-1 | LRA-12 |  |
| AIM-1 | 0 | 1.7262 | 1.6776 | 0.9005 | 1.6101 | 1.6415 | 1.4036 | 1.5861 |  |

The PDB IDs of the selected MBLs were as follows: AIM-1 (4P62), GOB-18 (5K0W), FEZ-1 (1K07), Rm3 (5IQK), SMB-1 (3VPE), L1 (2AIO), BJP-1 (5NJW), and LRA-12 (5AEB).

**Table S6.** The distance between the two zinc ions of the metallo-β-lactamases (MBLs)

|  |  | Chain A | Chain B | Chain C | Chain D |
| --- | --- | --- | --- | --- | --- |
| B1 | NDM-1 | 3.1 | 3.8 |  |  |
|  | BlaB-1 | 3.7 | 3.7 | 3.7 | 3.7 |
|  | VIM-2 | 3.5 |  |  |  |
|  | DIM-1 | 4.2 | 4.4 |  |  |
|  | IMP-1 | 3.4 | 3.4 | 3.3 | 3.4 |
|  | TMB-1 | 3.5 | 3.1 |  |  |
|  | SPS-1 | 3.5 |  |  |  |
|  | ECV-1 | 3.5 |  |  |  |
|  | MYO-1 | 3.7 | 3.5 |  |  |
|  | FIM-1 | Monozinc | | | |
|  | GIM-1 | 3.5 | 3.5 | 3.5 |  |
| B2 | CphA | Monozinc | | | |
|  | SfhI | Monozinc | | | |
| B3 | AIM-1 | 3.5 |  |  |  |
|  | GOB-18 | 3.5 | 3.8 |  |  |
|  | FEZ-1 | 3.7 | 3.7 |  |  |
|  | Rm3 | 3.5 | 3.5 |  |  |
|  | SMB-1 | 3.5 |  |  |  |
|  | L1 | 3.7 |  |  |  |
|  | BJP-1 | 3.4 | 3.4 |  |  |
|  | LRA-12 | 3.6 | 3.7 |  |  |
| Average |  | 3.56 | 3.6 | 3.5 | 3.6 |

The PDB IDs of the selected MBLs were as follows: NDM-1 (3S0Z), BlaB-1 (1M2X), VIM-2 (4NQ2), DIM-1 (4ZEJ), IMP-1 (5EV6), TMB-1 (5MMD), SPS-1 (6CQS), ECV-1 (6T5K), MYO-1 (6T5L), FIM-1 (6V3Q), GIM-1 (2YNT), CphA (1X8G), SfhI (5EW0), AIM-1 (4P62), GOB-18 (5K0W), FEZ-1 (1K07), Rm3 (5IQK), SMB-1 (3VPE), L1 (2AIO), BJP-1 (5NJW), and LRA-12 (5AEB).

**Table S7**. The inhibitory spectrum of known metallo-β-lactamase (MBL) inhibitors

| Inhibitor | | | Co-crystallized  MBL (PDB ID) | Inhibitory effects (IC_50_ or Ki in uM) | | | | | | Reference |
| --- | --- | --- | --- | --- | --- | --- | --- | --- | --- | --- |
| Inhibition type | Inhibitor name | Chemical structure |  | B1 | | B2 | | B3 | |  |
|  |  |  |  | IC_50_ (I) or  K_i_ (K) | Target | IC_50_ (I) or  K_i_ (K) | Target | IC_50_ (I) or  K_i_ (K) | Target |  |
| B1 MBL inhibitor | Cyclic boronate | 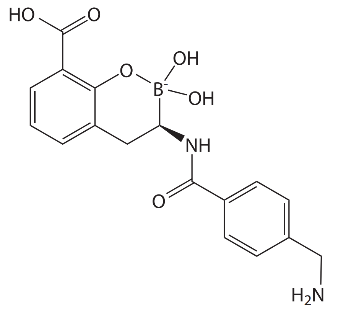 | VIM-2 (5FQC) | I, 0.3 | BcII | I, >100 | CphA | NA | NA | (Brem et al., 2016) |
|  |  |  |  | I, 0.003 | VIM-2 | NA | NA | NA | NA |  |
|  |  |  |  | I, 1.0 | IMP-1 | NA | NA | NA | NA |  |
|  |  |  |  | I, 0.029 | NDM-1 | NA | NA | NA | NA |  |
|  | Tricyclic natural product  (SB-236050) | 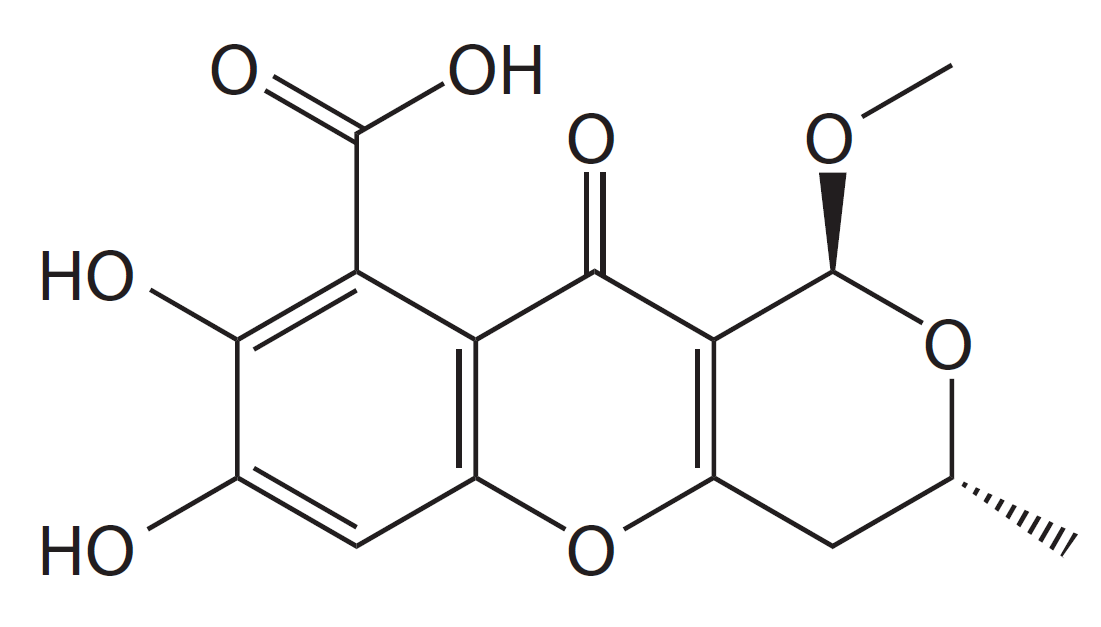 | CcrA (1KR3) | I, 29 | CcrA-1 | NA | NA | I, >1,000 | L-1 | (Payne et al., 2002) |
|  |  |  |  | I, 113 | IMP-1 | NA | NA | NA | NA |  |
|  | Benzophenone  [2-(4-fluorophenyl)carbonylbenzoic acid] | 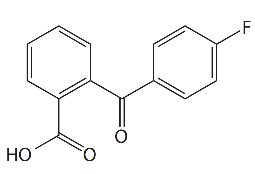 | VIM-2 (5ACX) | I, 14 | VIM-2 | NA | NA | NA | NA | (Christopeit et al., 2015) |
|  | Isoquinoline  [2-(3-fluoro-4-hydroxyphenyl)-3-oxoisoindoline-4-carboxylic acid] | 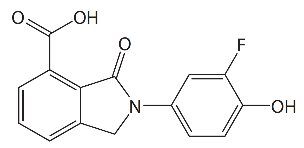 | VIM-2 (5LM6) | I,7.7 | VIM-2 | NA | NA | NA | NA | (Li et al., 2017) |
|  |  |  |  | I, >400 | VIM-5 | NA | NA | NA | NA |  |
|  |  |  |  | I, >400 | VIM-1 | NA | NA | NA | NA |  |
|  | Benzyl thiol  [4-phenyl-2-(sulfanylmethyl)benzoic acid] | 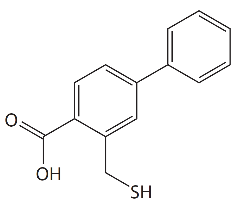 | VIM-2 (5K48) | I, 5.59 | NDM-1 | NA | NA | NA | NA | (Cain et al., 2018) |
|  |  |  |  | I, 0.23 | VIM-2 | NA | NA | NA | NA |  |
|  |  |  |  | I, 0.23 | IMP-1 | NA | NA | NA | NA |  |
|  | Disubstituted succinic acid  [2,3-bis-benzo(1,3)dioxol-5-ylmethyl-succinic acid] | 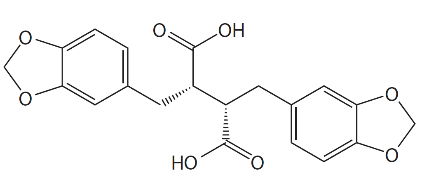 | IMP-1 (1JJT) | I, 0.009 | IMP-1 | NA | NA | NA | NA | (Toney et al., 2001) |
|  | Biphenyl tetrazole  [ L-159,061; 2-Butyl-6-hydroxy-3-[2'-(1H-tetrazol-5-yl)-biphenyl-4-ylmethyl]-3H-quinazolin-4-one] | 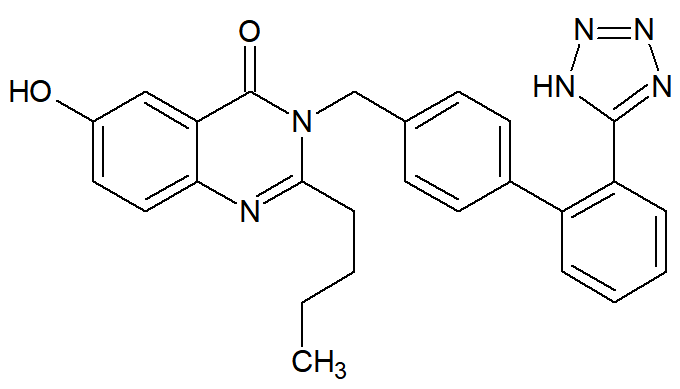 | CcrA (1A8T) | I, 1.9 | CcrA | NA | NA | NA | NA | (Toney et al., 1998) |
| Cross-class MBL inhibitor | Bisthiazolidine  (L-VC26) | 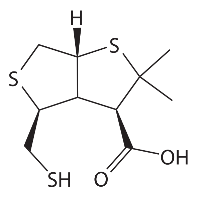 | IMP-1 (5EWA) | I, 18 | NDM-1 | NA | NA | I, 11 | L1 | (Hinchliffe et al., 2016) |
|  |  |  |  | I, 6 | VIM-2 | NA | NA | I, 31 | GOB-18 |  |
|  |  |  |  | I, 15 | IMP-1 | NA | NA | NA | NA |  |
|  |  |  |  | I, 32 | BcII | NA | NA | NA | NA |  |
|  | R-Thiomandelic acid | 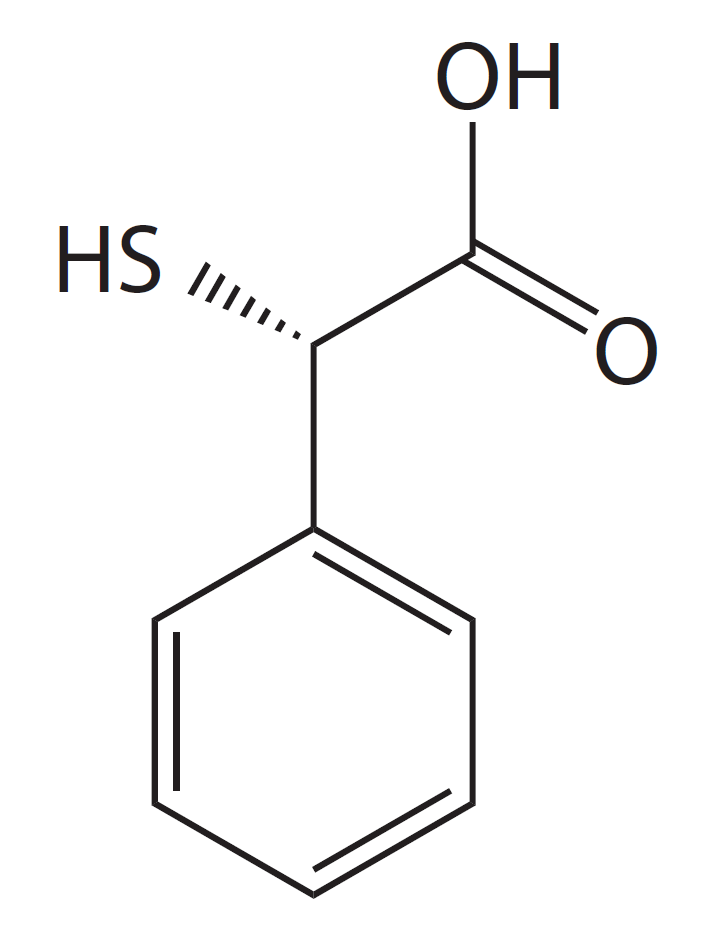 | BCII (2M5D) | K, 0.34 | BcII | K, 144 | CphA | K, 0.081 | L1 | (Karsisiotis et al., 2013; Mollard et al., 2001) |
|  |  |  |  | K, 0.8 | CcrA | NA | NA | K, 0.27 | FEZ-1 |  |
|  |  |  |  | K, 0.059 | IMP-1 | NA | NA | NA | NA |  |
|  |  |  |  | K, 0.23 | VIM-1 | NA | NA | NA | NA |  |
|  |  |  |  | K, 0.56 | BlaB | NA | NA | NA | NA |  |
|  | Thiol-containing derivative  [(R)-(2,4-dichlorophenyl)(sulfanyl)methyl]phosphonic acid | 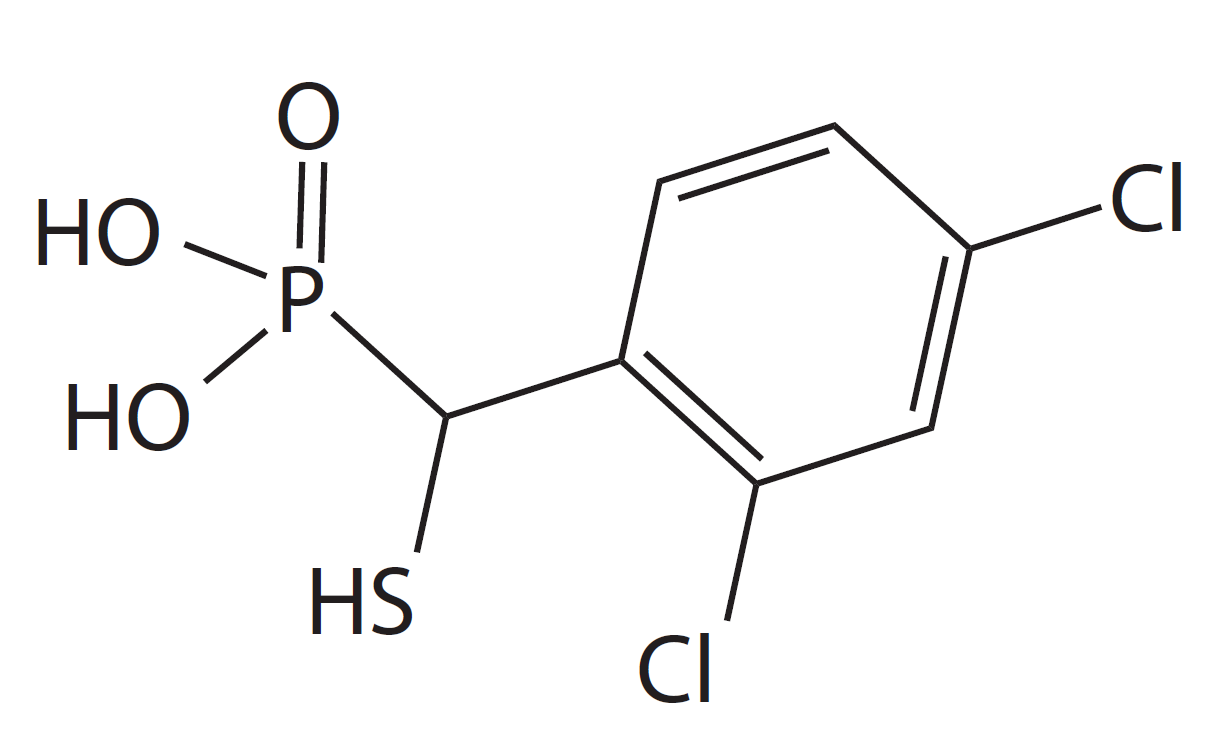 | CphA (3IOG) | K, 1 | VIM-4 | K, 5 | CphA | K, 0.4 | L1 | (Lassaux et al., 2010) |
|  | Thiol-containing derivative  {bis(1-methylethyl) [2-(sulfanylmethyl)phenyl]phosphonate} | 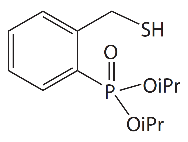 | CphA (3IOF) | K, 3 | VIM-4 | K, 2 | CphA | K, 3 | L1 | (Lassaux et al., 2010) |

* NA means these data are not available.
